# Effect of broccoli sprout extract and baseline gut microbiota on fasting blood glucose in prediabetes: a randomized, placebo-controlled trial

**DOI:** 10.1038/s41564-025-01932-w

**Published:** 2025-02-10

**Authors:** Chinmay Dwibedi, Annika S. Axelsson, Birgitta Abrahamsson, Jed W. Fahey, Olof Asplund, Ola Hansson, Emma Ahlqvist, Valentina Tremaroli, Fredrik Bäckhed, Anders H. Rosengren

**Affiliations:** 1 Department of Neuroscience and Physiology, Sahlgrenska Academy, University of Gothenburg, Gothenburg, Sweden; 2 The Wallenberg Laboratory, Department of Molecular and Clinical Medicine, Sahlgrenska Academy, University of Gothenburg, Gothenburg, Sweden; 3 Department of Clinical Microbiology and Molecular Infection Medicine Sweden (MIMS), Umeå University, Umeå, Sweden; 4 Departments of Medicine, Pharmacology and Molecular Sciences, and Psychiatry and Behavioral Sciences, Johns Hopkins University, Baltimore, MD USA; 5 Department of Clinical Sciences, Lund University Diabetes Center, Lund University, Malmö, Sweden; 6 Institute for Molecular Medicine Finland, Helsinki University, Helsinki, Finland; 7 Region Västra Götaland, Department of Clinical Physiology, Sahlgrenska University Hospital, Gothenburg, Sweden

**Keywords:** Pre-diabetes, Pre-diabetes

## Abstract

More effective treatments are needed for impaired fasting glucose or glucose intolerance, known as prediabetes. Sulforaphane is an isothiocyanate that reduces hepatic gluconeogenesis in individuals with type 2 diabetes and is well tolerated when provided as a broccoli sprout extract (BSE). Here we report a randomized, double-blind, placebo-controlled trial in which drug-naive individuals with prediabetes were treated with BSE (*n* = 35) or placebo (*n* = 39) once daily for 12 weeks. The primary outcome was a 0.3 mmol l^−1^ reduction in fasting blood glucose compared with placebo from baseline to week 12. Gastro-intestinal side effects but no severe adverse events were observed in response to treatment. BSE did not meet the prespecified primary outcome, and the overall effect in individuals with prediabetes was a 0.2 mmol l^−1^ reduction in fasting blood glucose (95% confidence interval −0.44 to −0.01; *P* = 0.04). Exploratory analyses to identify subgroups revealed that individuals with mild obesity, low insulin resistance and reduced insulin secretion had a pronounced response (0.4 mmol l^−1^ reduction) and were consequently referred to as responders. Gut microbiota analysis further revealed an association between baseline gut microbiota and pathophysiology and that responders had a different gut microbiota composition. Genomic analyses confirmed that responders had a higher abundance of a *Bacteroides*-encoded transcriptional regulator required for the conversion of the inactive precursor to bioactive sulforaphane. The abundance of this gene operon correlated with sulforaphane serum concentration. These findings suggest a combined influence of host pathophysiology and gut microbiota on metabolic treatment response, and exploratory analyses need to be confirmed in future trials. ClinicalTrials.gov registration: NCT03763240.

## Abstract (web summary)

In a randomized controlled trial, the effect of sulforaphane (provided as a broccoli sprout extract) on fasting blood glucose in individuals with prediabetes was tested, in the context of gut microbiota composition and function.

## Main

While many resources are devoted to treating late stages of type 2 diabetes, when anti-hyperglycaemic therapy usually has limited effect on disease progression, individuals with prediabetes, who have the greatest opportunities for reversal^1–4^, only occasionally receive structured treatment^5^. International guidelines recommend lifestyle intervention programmes for individuals with prediabetes^5^, but these are resource intense, have variable outcomes and are currently offered to less than 10% of individuals with increased blood glucose^6^. Obese individuals below 60 years of age, individuals with other high-risk traits or women with previous gestational diabetes who fail to improve glycaemic control through lifestyle changes are recommended additional metformin treatment^5^. However, several countries, including many European countries, do not routinely treat prediabetes pharmacologically because of the associated side effects (including detrimental effects on gut microbiota and host metabolism)^7,8^ and the large variation in metabolic response (for example, 30% do not respond to metformin)^9,10^. To enable more versatile and personalized prevention, it is therefore important to investigate other treatment options, including nutritional supplements that may improve glucose control^11,12^.

Current guidelines also emphasize the need to investigate treatment efficacy in different subgroups of patients^8^. Several methods to stratify patients into subgroups (based on pathophysiology or genetics) have been proposed, but the relevance of such stratification in predicting treatment response is largely unknown^8,13–16^. Moreover, recent studies have shown a potential role of the gut microbiome in the progression of diabetes and the response to anti-diabetic treatment. It has been shown that the gut microbiome is changed in individuals with prediabetes and diabetes, with decreased abundance of butyrate producers, compared with normoglycaemic individuals^17^. In addition, treatment with anti-diabetic drugs such as metformin has been associated with consistent shifts in microbial functions, including the biosynthesis of lipopolysaccharides and the metabolism of short-chain fatty acids^18^. Furthermore, the gut microbiome plays an important role in metabolizing dietary nutrients in the host. It could therefore potentially influence the glycaemic response to nutritional interventions^19,20^. Several gaps remain, however, in our understanding of how both the individual gut microbiota and pathophysiology affect the glycaemic treatment response, in particular in prediabetes^21–23^.

We have recently found that sulforaphane, an isothiocyanate previously studied for cancer prevention^11^, reduces hepatic glucose production, as verified in both animal models and patients with obesity and dysregulated type 2 diabetes^12^. The mechanism of action was shown to involve nuclear translocation of nuclear factor erythroid 2-related factor 2 (NRF2), resulting in decreased expression of gluconeogenic enzymes, including phosphoenolpyruvate carboxykinase (PEPCK)^12^. Interestingly, the biogenic precursor to sulforaphane, the glucosinolate glucoraphanin, is contained at high concentrations in cruciferous vegetables, such as broccoli, and when provided as a broccoli sprout extract (BSE), delivering 150 μmol sulforaphane per dose, glucose tolerance was improved to the same extent as by pure (99% reagent-grade) sulforaphane. Ablation of sulforaphane in the BSE abolished the effect, showing that sulforaphane is the active component^12^. The action of the compound on hepatic gluconeogenesis, its high tolerability^11^ and the ability to provide the compound as a BSE, making it available at a very low cost per dose, make its investigation as a possible anti-hyperglycaemic treatment at prediabetic stages highly warranted.

We hypothesized that BSE can be effective as an early intervention in treatment-naive individuals with impaired fasting blood glucose and tested this hypothesis in a double-blind randomized trial. The rationale for focusing on individuals with impaired fasting glucose is that sulforaphane directly suppresses hepatic gluconeogenesis^12^. This is central to the pathophysiology of impaired fasting glucose, in contrast to the larger involvement of peripheral insulin resistance in impaired glucose tolerance^2,8^. The primary endpoint was the change in fasting blood glucose from baseline in participants assigned to BSE compared with placebo. The effect of BSE was also studied in different pathophysiological subgroups. Thus, in a post hoc exploratory analysis, we stratified study participants using a data-driven clustering algorithm that has been reproduced across multiple diabetic cohorts^14–16^ and tested the null hypothesis that treatment response does not differ between subgroups. Finally, we analysed the microbiota composition in the subgroups and its association with response to BSE.

## Results

### Patient characteristics and safety

The inclusion criteria were impaired fasting glucose (6.1–6.9 mmol l^−1^), 35–75 years of age, a body mass index (BMI) of 27–45 kg m^−2^ and written informed consent. Individuals with conditions or treatments that may affect blood glucose were excluded (see Methods for full study criteria). Impaired fasting glucose was defined using the international World Health Organization criteria rather than the wider criteria proposed by the American Diabetes Association, as it enabled the investigation of individuals with more severe disease progression and higher risk for adverse outcomes, who are likely to benefit more from therapeutic interventions^8^. A total of 450 individuals were screened for impaired fasting glucose, of whom 89 individuals met the study criteria and were included. The included individuals had a mean age of 63 ± 9 years and 64% were men. The average fasting blood glucose was 6.4 ± 0.2 mmol l^−1^, and the average BMI was 32 ± 4 kg m^−2^.

**Fig. 1:**
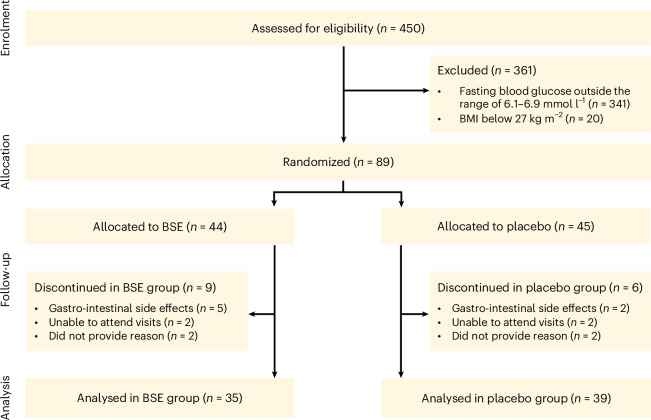
Study profile as a CONSORT diagram. Number of individuals randomized and assigned to BSE and placebo, respectively. In addition to the reasons provided for study discontinuation, a full list of reported adverse events for all participants is presented in Table 2. Source data

The participants were randomized to receive sulforaphane-containing BSE (150 μmol once daily) or placebo for 12 weeks (Table 1). A total of 15 participants were lost to full clinical follow-up, mainly because of gastro-intestinal side effects (9 assigned to BSE and 6 to placebo; Fig. 1 and Supplementary Table 1). A higher frequency of gastro-intestinal side effects, including loose stools, nausea, diarrhoea, vomiting and reflux, was reported in participants receiving BSE than in those receiving placebo, and those who discontinued had a higher frequency of gastro-intestinal side effects than participants who completed the study, particularly those in the BSE-treated group (Table 2).

**Table 1:** Demographic and baseline characteristics of participants in the study

Characteristics	BSE (*n* = 44)	Placebo (*n* = 45)	All (*n* = 89)
Age (years)	65 ± 7	61 ± 10	63 ± 9
Male (*n* (%))	29 (66)	28 (62)	57 (64)
Fasting glucose (mmol l^−1^)	6.4 ± 0.2	6.4 ± 0.2	6.4 ± 0.2
HbA1c (mmol mol^−1^)	38.2 ± 3.9	38.1 ± 4.5	38.2 ± 4.1
BMI^a^	32.1 ± 3.8	32.2 ± 3.9	32.1 ± 3.8
HOMA-B	118.1 ± 30.0	119.1 ± 28.1	118.6 ± 28.9
HOMA-IR	5.4 ± 3.2	5.4 ± 3.4	5.4 ± 3.3
Fasting insulin (mIE l^−1^)	18.8 ± 10.8	19.0 ± 11.5	18.9 ± 11.1
Fasting C-peptide (nmol l^−1^)	1.22 ± 0.45	1.22 ± 0.39	1.22 ± 0.42
Fasting C-peptide-to-insulin ratio	0.074 ± 0.022	0.073 ± 0.020	0.074 ± 0.021
Bilirubin (μmol l^−1^)	10.0 ± 4.1	9.8 ± 4.5	9.9 ± 4.3
ALP (μkat l^−1^)	1.1 ± 0.3	1.1 ± 0.3	1.1 ± 0.3
GGT (μkat l^−1^)	0.7 ± 0.4	0.7 ± 0.5	0.7 ± 0.5
AST (μkat l^−1^)	0.4 ± 0.2	0.5 ± 0.2	0.4 ± 0.2
ALT (μkat l^−1^)	0.5 ± 0.3	0.6 ± 0.3	0.6 ± 0.3
Fatty liver index^b^	75.9 ± 20.5	76.9 ± 17.4	76.4 ± 18.9
Total cholesterol (mmol l^−1^)	5.0 ± 1.1	5.1 ± 1.0	5.1 ± 1.0
LDL (mmol l^−1^)	3.4 ± 1.0	3.5 ± 1.0	3.5 ± 1.0
HDL (mmol l^−1^)	1.4 ± 0.4	1.3 ± 0.4	1.4 ± 0.4
Triglycerides (mmol l^−1^)	1.4 ± 0.6	1.5 ± 0.6	1.5 ± 0.6
Creatinine (μmol l^−1^)	82.1 ± 16.3	74.9 ± 12.5	78.5 ± 14.9
Estimated glomerular filtration rate (ml min^−1^ 1.73 m^−^^2^)	71.4 ± 11.0	78.8 ± 10.2	75.1 ± 11.1
Baseline physical activity (metabolic minutes per week)^c^	2410 ± 1414	2225 ± 2028	2317 ± 1704
Food frequency score^d^	4.7 ± 1.3	4.7 ± 2.3	4.7 ± 1.8

Plus–minus values are means ± s.d. LDL, low-density lipoprotein; HDL high-density lipoprotein.

^a^The BMI is the weight in kilograms divided by the square of the height in metres.

^b^The fatty liver index was calculated based on BMI, waist circumference, triglycerides and GGT.

^c^Self-reported data via the IPAQ.

^d^Food frequency questionnaire score from 0 to 9 (with 9 indicating a diet most adherent to official food recommendations) as described in Methods.

Source data

**Table 2:** Reported adverse events in study participants

Adverse events	Full analysis set (*n* = 74)			Discontinued (*n* = 15)			All (*n* = 89)		
	Placebo (*n* = 39)	BSE (*n* = 35)	Total (*n* = 74)	Placebo (*n* = 6)	BSE (*n* = 9)	Total (*n* = 15)	Placebo (*n* = 45)	BSE (*n* = 44)	Total (*n* = 89)
Nausea		3	3		2	2		5	5
Loose stools		6	6	1	2	3	1	8	9
Diarrhoea	1	1	2	1	3	4	2	4	6
Vomiting	1	1	2		1	1	1	2	3
Gastro-intestinal reflux	1	1	2				1	1	2
Lower urinary tract infection	1		1				1		1
Upper urinary tract infection	1		1				1		1
Pneumonia		1	1					1	1
Upper respiratory tract infection	3	5	8	1	1	2	4	6	10
Dry mouth	1		1				1		1
Fainting		1	1					1	1
Tooth infection	1		1				1		1
Hot flush	1		1				1		1
Skin symptoms	1		1				1		1
Skeletal pain	3	2	5				3	2	5
Gout		1	1					1	1

Adverse events were reported at the final visit or during a telephone follow-up. Data from discontinued participants are only from telephone follow-up, as they did not attend the final visit. Each participant may report several adverse events. Nausea, loose stools, diarrhoea, vomiting and gastro-intestinal reflux are collectively referred to as gastro-intestinal side effects in the text.

Source data

### Primary analysis of fasting blood glucose

The participants who were assigned to BSE had a larger average reduction of fasting blood glucose than those receiving placebo, with a mean difference of 0.2 mmol l^−1^ between the randomization groups (95% confidence interval (CI) −0.44 to −0.01; *P* = 0.04 using a linear model adjusted for BMI and variation in homeostasis model assessment estimates of insulin resistance (HOMA-IR); *P* = 0.045 using ANCOVA; Table 3 and Supplementary Table 2). However, this did not meet the prespecified outcome of 0.3 mmol l^−1^ mean difference between randomization groups, which was set based on the efficacy previously observed in patients with type 2 diabetes^12^.

**Table 3:** Effect of BSE on primary and secondary endpoints

Endpoint	Mean difference (95% CI)^a^
Change in fasting glucose (mmol l^−1^)	−0.2 (−0.44 to −0.01)
Change in HbA1c (mmol mol^−1^)	−0.3 (−1.3 to 0.6)
Change in BMI^b^	−0.4 (−0.8 to 0.0)
Change in HOMA-B	7.1 (−3.2 to 17.4)
Change in HOMA-IR	0.9 (−0.1 to 1.9)
Change in fasting insulin (mIE l^−1^)	3.4 (0.1 to 6.8)
Change in fasting C-peptide (nmol l^−1^)	0.07 (−0.02 to 0.17)
Change in fasting C-peptide-to-insulin ratio	−0.006 (−0.016 to 0.005)
Change in fatty liver index^c^	−1.2 (−5.2 to 2.9)
Change in total cholesterol (mmol l^−1^)	0.1 (−0.2 to 0.3)
Change in LDL cholesterol (mmol l^−1^)	0.0 (−0.2 to 0.2)
Change in HDL cholesterol (mmol l^−1^)	0.1 (−0.0 to 0.1)
Change in triglycerides (mmol l^−1^)	0.1 (−0.1 to 0.4)
Change in physical activity (metabolic minutes per week)^d^	737 (−1,702 to 3,176)
Change in food frequency score^e^	−0.02 (−0.99 to 0.95)

Changes relative to the baseline in primary and secondary endpoints in response to the BSE or placebo, respectively, in the full analysis set (*n* = 35 assigned to BSE; *n* = 39 assigned to placebo).

^a^Estimated mean differences of values in response to BSE minus placebo are presented as means with 95% CIs.

^b^The BMI is the weight in kilograms divided by the square of the height in metres.

^c^The fatty liver index was calculated based on BMI, waist circumference, triglycerides and GGT.

^d^Self-reported data via the IPAQ.

^e^Food frequency questionnaire score from 0 to 9 (with 9 indicating a diet most adherent to official food recommendations) as described in Methods.

Source data

### Analysis of secondary outcomes

**Extended Data Fig. 1:**
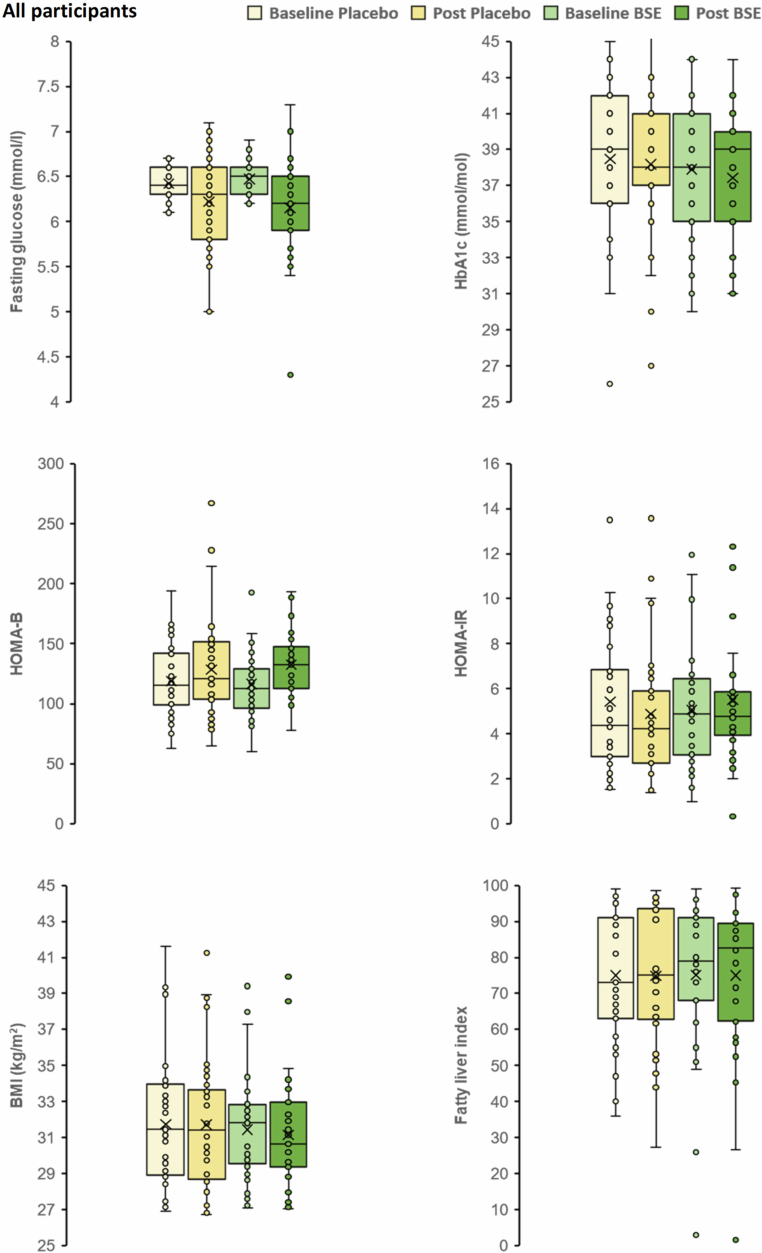
Distribution of primary and secondary variables in all participants. Box plots show individual data points, medians (straight lines inside box) and means (cross marking) with hinges representing lower and upper quartile. Data are from baseline and post-treatment in the placebo (n = 39) and BSE (n = 35) groups. Individuals with similar values are represented by the same circle. Source data

**Extended Data Fig. 2:**
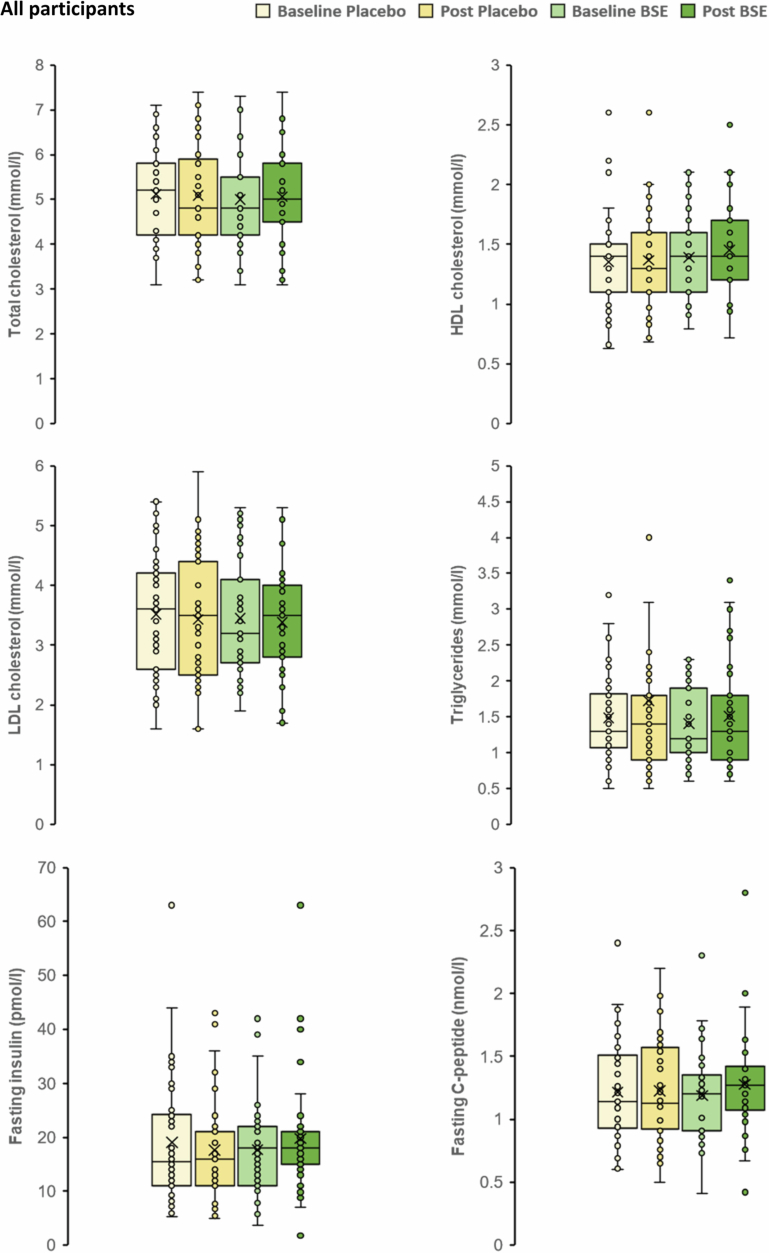
Distribution of cholesterol, triglycerides, insulin and C-peptide in all participants. Box plots show individual data points, medians (straight lines inside box) and means (cross marking) with hinges representing lower and upper quartile. Data are from baseline and post-treatment in the placebo (n = 39) and BSE (n = 35) groups. Individuals with similar values are represented by the same circle. Source data

There was no difference in the change of BMI, HOMA-IR, HOMA estimate of beta-cell function (HOMA-B; reflecting insulin secretion), glycated haemoglobin (HbA1c), insulin clearance, fatty liver index, plasma cholesterol, serum triglyceride concentration, physical activity or dietary pattern between the groups (Table 3 and Extended Data Figs. 1 and 2).

### Exploratory analysis of pathophysiological subgroups

**Fig. 2:**
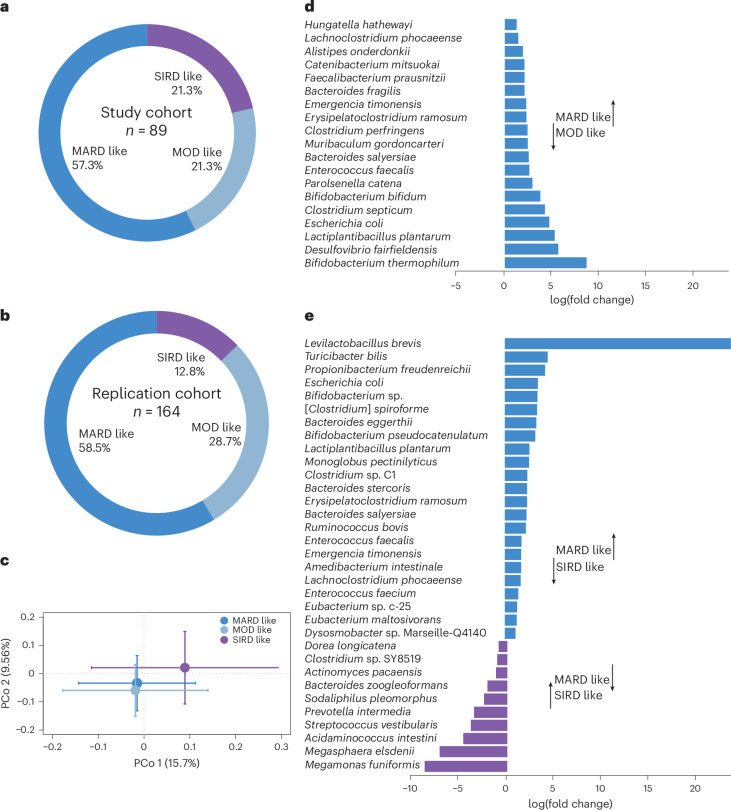
Participant distribution and gut microbiota composition in clusters. **a**, Distribution of study participants in pathophysiological clusters according to the clustering methodology in ref. ^14^ (*n* = 89). **b**, Cluster distribution of participants in the replication cohort (*n* = 164). **c**, PCo analysis of Bray–Curtis dissimilarities at the species level in the three clusters of study participants (*P* = 0.02; *n* = 67 samples from 15 participants with SIRD-like characteristics, 11 with MOD-like characteristics and 41 with MARD-like characteristics). Standard error bars for the principal coordinates are denoted. **d**,**e**, Significantly altered taxa (at adjusted *P* < 0.05) in the microbiota between participants with MARD-like and MOD-like characteristics (**d**) and between participants with MARD-like and SIRD-like characteristics (**e**). The *x* axis denotes log(fold change) of the taxa in the cluster with MARD-like characteristics compared with the clusters with MOD-like and SIRD-like characteristics, respectively, as indicated. *Clostridium spiroforme* is in the process of getting renamed and therefore within brackets. The legend in **c** also applies to **d** and **e**. Source data

We next investigated whether specific clinical and pathophysiological characteristics were associated with the metabolic response to BSE by post hoc exploratory analyses. A data-driven cluster analysis of newly diagnosed patients has recently identified five subgroups of diabetes with different clinical features, pathophysiology and disease progression^14^. We extended this approach to prediabetes and observed that 19 study participants (10 BSE, 9 placebo) had early but typical characteristics of severe insulin-resistant diabetes (SIRD), a subgroup that features high BMI and insulin resistance and has been associated with increased prevalence of non-alcoholic fatty liver disease (NAFLD)^15^ (Supplementary Table 3). Another 19 participants (5 BSE, 14 placebo) had features corresponding to mild obesity-related diabetes (MOD), with increased BMI and moderate insulin resistance. Finally, 51 study participants (29 BSE, 22 placebo) had the typical characteristics of mild age-related diabetes (MARD), with comparatively low BMI, insulin resistance, fatty liver index and insulin secretion (Fig. 2a). To assess the stability and reproducibility of the cluster distribution in prediabetes, we also used data from an independent cohort of 164 individuals with impaired fasting blood glucose (Fig. 2a,b, Supplementary Tables 4 and 5, and Supplementary Notes).

**Extended Data Fig. 3:**
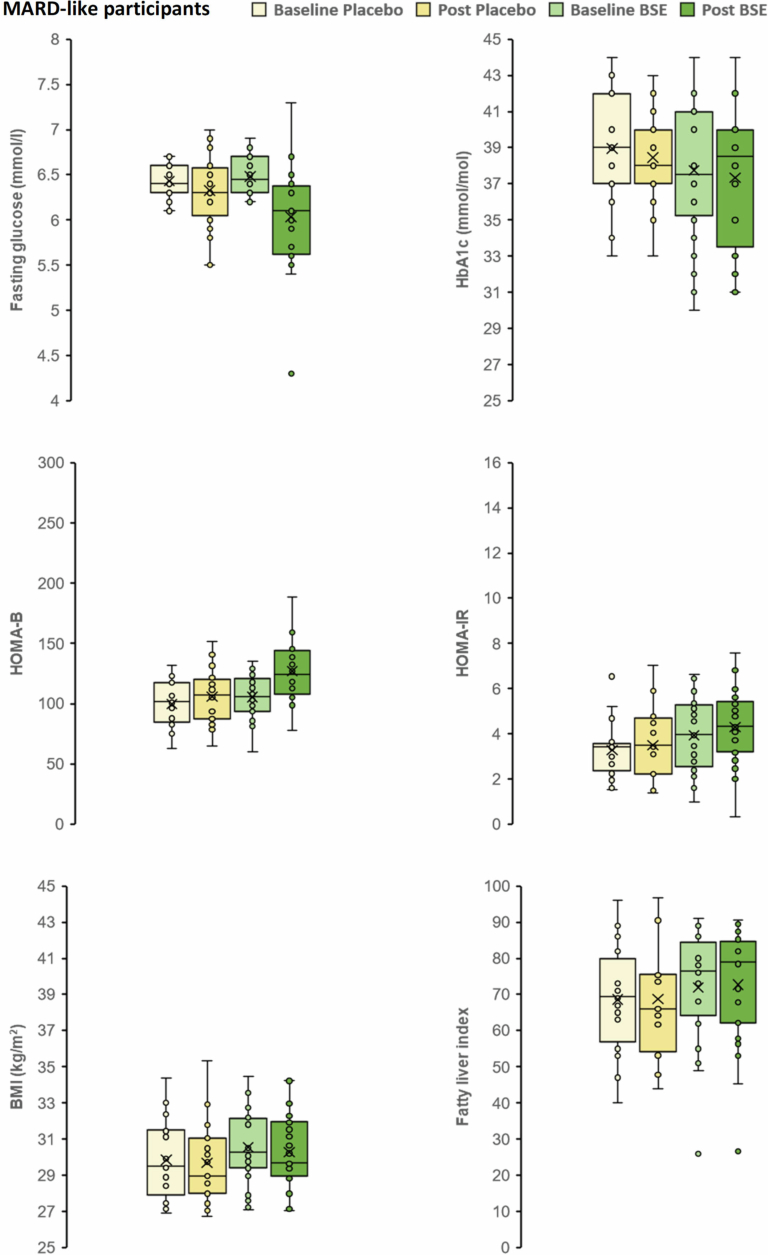
Distribution of primary and secondary variables in MARD-like participants. Box plots show individual data points, medians (straight lines inside box) and means (cross marking) with hinges representing lower and upper quartile. Data are from baseline and post-treatment in the placebo (n = 20) and BSE (n = 24) groups of MARD participants. Individuals with similar values are represented by the same circle. Source data

**Extended Data Fig. 4:**
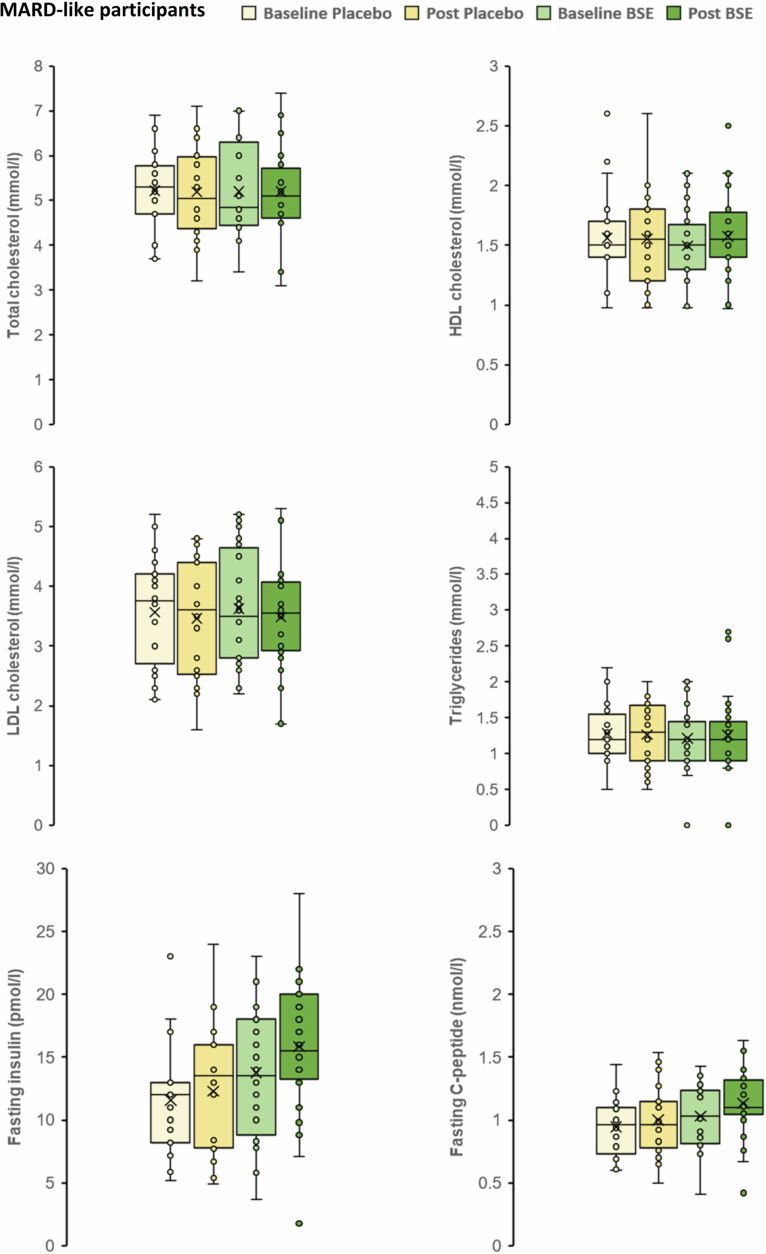
Distribution of cholesterol, triglycerides, insulin and C-peptide in MARD-like participants. Box plots show individual data points, medians (straight lines inside box) and means (cross marking) with hinges representing lower and upper quartile. Data are from baseline and post-treatment in the placebo (n = 20) and BSE (n = 24) groups of MARD participants. Individuals with similar values are represented by the same circle. Source data

The response to BSE (change in fasting glucose relative to baseline) differed between participants of the various clusters (Supplementary Table 6). Those in the cluster with MARD-like characteristics had a greater improvement of fasting blood glucose in response to treatment, with a mean difference of 0.4 mmol l^−1^ between BSE and placebo (95% CI −0.6 to −0.1; *n* = 24 with BSE and *n* = 20 with placebo; *P* = 0.008; Extended Data Figs. 3 and 4). Moreover, they had improved insulin secretion, measured as HOMA-B (95% CI 3.3–26.0; *P* = 0.02). By contrast, there was no significant difference between BSE and placebo in participants in the clusters with SIRD- or MOD-like characteristics (Supplementary Figs. 1–4). When evaluated as an interaction term, treatment (BSE or placebo) and cluster were observed to have a statistically significant interaction (*P* = 0.008 using a linear model, with the change in fasting glucose as the dependent variable; *n* = 74; Supplementary Table 6). This suggests that the glycaemic response to BSE differs based on cluster.

### Exploratory analysis of gut microbiota in responders

**Extended Data Fig. 5:**
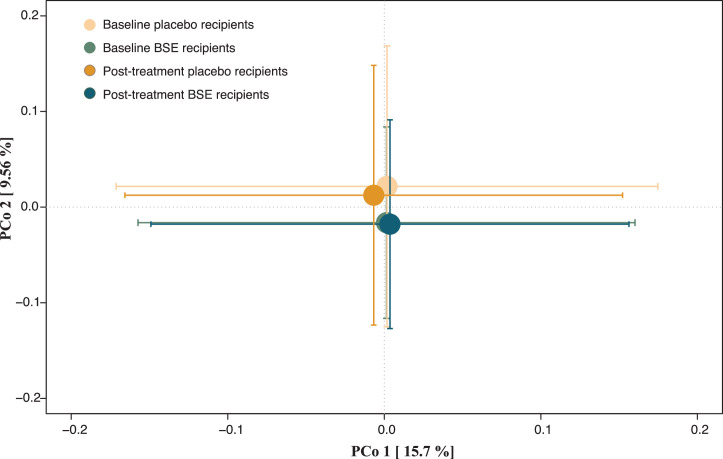
Dissimilarities between BSE and placebo. Principal coordinates analysis of species-level Bray-Curtis dissimilarities at baseline and post-treatment between study participants randomized to BSE and placebo, respectively. No significant differences were observed. Source data

We also obtained stool samples before and after treatment with BSE or placebo for whole-genome sequencing. Principal coordinate (PCo) analysis of Bray–Curtis dissimilarity at the species level showed similar gut microbiota composition at baseline in the randomization groups, with no compositional change in response to treatment (Extended Data Fig. 5). Interestingly, we observed significant differences in overall baseline microbiota composition between participants of the clinical clusters (*P* = 0.02; Fig. 2c). Compared with the other subgroups, the gut microbiota of participants with MARD characteristics had an increased abundance of health-associated species, such as those in *Bifidobacterium*, *Levilactobacillus* and *Lactiplantibacillus*, and butyrate producers such as *Faecalibacterium* and *Eubacterium*^24,25^ (Fig. 2d,e). This microbiota composition reflects the milder clinical phenotype of the cluster with MARD-like characteristics (lower BMI, HOMA-IR and fatty liver index) compared with the other clusters^24,25^.

**Fig. 3:**
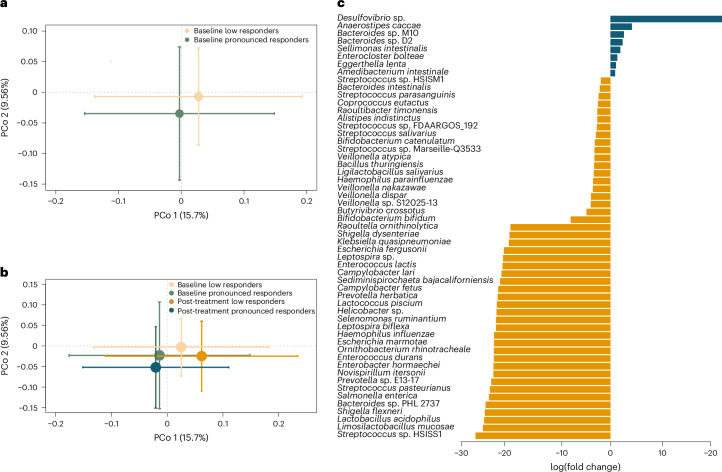
Gut microbiota profiles of participants with different responses to BSE. **a**, PCo analysis of Bray–Curtis dissimilarities of gut microbiota at the species level at baseline between participants who showed a pronounced response (defined as a reduction of fasting blood glucose of at least 0.3 mmol l^−1^; *n* = 13) and a less pronounced response (*n* = 22) to BSE treatment (*P* = 0.001). Standard error bars for the principal coordinates are denoted. **b**, PCo analysis of species‐level Bray–Curtis dissimilarities at baseline and post-treatment in participants with a pronounced (*n* = 13) and less pronounced (*n* = 22) response to BSE (no significant differences between baseline and post-treatment; standard error bars are denoted). **c**, Significantly altered taxa at baseline (at adjusted *P* < 0.05) between participants with a pronounced (blue) and less pronounced (yellow) response to BSE. The *x* axis denotes log(fold change) of the taxa. Source data

Although participants with MARD-like characteristics had a larger average glycaemic response to BSE than those with SIRD- and MOD-like characteristics, all individuals with MARD-like characteristics did not respond equally well (Extended Data Fig. 3). To further understand which factors, in addition to pathophysiological cluster, influence the glycaemic response, we contrasted, in a post hoc analysis, the participants who showed a pronounced response to BSE (defined as a reduction of fasting blood glucose greater than the top quartile of glycaemic improvement (0.3 mmol l^−1^); *n* = 13) with the remainder (*n* = 22). Of the 13 pronounced responders, 11 were in the cluster with MARD-like characteristics, further corroborating that MARD characteristics are important for the treatment response. Moreover, we observed that the baseline gut microbiota composition of the pronounced responders was significantly different (*P* = 0.001) from that of the remainder (Fig. 3a), without further changes after treatment (Fig. 3b).

In particular, the metagenome of pronounced responders had increased abundance of *Desulfovibrio* sp., *Anaerostipes caccae*, *Bacteroides* M10 and *Bacteroides* D2 (Fig. 3c). *Desulfovibrio* sp. maintains gut fermentative processes by removing electron sink by-products of fermentation, such as lactate and hydrogen, and they have recently been shown to use lactate and support the growth of butyrate-producing bacteria such as *Faecalibacterium prausnitzii*^26^. *A. caccae* is a known butyrate producer, and the observed shifts in microbial species were paralleled by increased abundance in pronounced responders of butyrate kinase (*P* = 0.047), one of the terminal enzymes catalysing butyrate production from carbohydrates (Supplementary Fig. 5)^27^.

By contrast, participants with a less pronounced response had significantly higher abundance of lactate producers, with increased facultative anaerobes and oral pathogens, including members of *Streptococcus* and *Veillonella* (Fig. 3c). These have previously been observed in individuals with metabolic disease, in particular NAFLD, who have lower colonization resistance against oral and opportunistic pathogens^28^. The observations are consistent with the increased plasma concentration of clinical markers of NAFLD in those with a less pronounced response, predominantly gamma-glutamyl transferase (GGT; mean difference 0.3 μkat l^−1^ (95% CI 0.1–0.6) compared with the pronounced responders). The lower abundance of butyrate producers and lower butyrate production potential in low responders are also characteristic of NAFLD (Supplementary Fig. 5)^29^. In addition, participants with a less pronounced response had higher gut microbial gene richness at baseline (*P* = 0.02; Supplementary Fig. 5), possibly reflecting the increased abundance of facultative anaerobes with longer genomes (Fig. 3c). While the gene richness in the pronounced responders was lower at baseline, it tended to be more homogenous after treatment (Supplementary Fig. 6), and the Bray–Curtis dissimilarity between samples decreased after treatment in the pronounced responders (*P* = 0.02; Supplementary Fig. 5).

### Exploratory analysis of the BT2160 operon in responders

Interestingly, *Bacteroides* M10 and *Bacteroides* D2 (Fig. 3c), which were both increased in the pronounced responders, have recently been shown to convert inactive glucosinolate to bioactive isothiocyanates such as sulforaphane^30^. We therefore explored these in further detail. In the two bacterial genomes, we identified the operon BT2156–BT2160, which plays an important role in glucosinolate conversion^30^, with gene length coverage of 99–100% and a high sequence similarity of 84–86%. Next, we analysed the distribution of the transcriptional regulator of the operon, BT2160, in the metagenome and found it to be significantly higher in the pronounced responders at baseline compared with the remainder (*P* = 0.02; Supplementary Fig. 5a), suggesting an increased potential for glucosinolate activation in this group.

We also identified several other genes that differed significantly between pronounced responders and the remainder (Supplementary Fig. 7). These genes are associated with various metabolic pathways, including sugar metabolism (for example, d-arabinitol 2-dehydrogenase), polyamine metabolism (for example, l-2,4-diaminobutyrate decarboxylase), amino acid metabolism, oxidative stress (for example, ATP-dependent RNA helicase), and cellular energy metabolism and mitochondrial function (for example, phosphate transport)^31^. While these genes indirectly relate to the pathogenesis of type 2 diabetes^31^, their specific influence on the fasting glucose concentration warrants further studies.

### Exploratory analysis of BT2160 and the efficacy of BSE

To further investigate the influence of BT2160 on the clinical effect of BSE, we analysed sulforaphane concentration in serum after BSE treatment. The sulforaphane concentration had a bimodal distribution in the BSE-treated participants (with a mean concentration of 0.2 nmol ml^−1^ at the low end and 1.3 nmol ml^−1^ at the high end and the mean values differing by approximately twice the common standard deviation; Supplementary Fig. 8). Interestingly, the participants with high sulforaphane concentration had significantly increased abundance of BT2160 in the gut microbiota compared with those with low sulforaphane concentration (0.032 versus 0.022; *P* = 0.046). The abundance of BT2160 did not differ between pathophysiological clusters. We observed, however, that in participants with MARD-like characteristics, the abundance of BT2160 was significantly correlated with the glycaemic response (*P* = 0.026; Spearman correlation coefficient −0.47). Moreover, when evaluated as an interaction term in the statistical model, there was a significant interaction between pathophysiological cluster (MARD-like versus SIRD- and MOD-like) and abundance of BT2160 on the glycaemic response to BSE (*P* = 0.048 for the interaction term; Supplementary Table 6 and Supplementary Fig. 5a).

We next contrasted participants who had an abundance of BT2160 below and above the median, respectively, and compared the glycaemic response between pathophysiological clusters in each stratum of BT2160 abundance. In participants with BT2160 abundance above the median, those with MARD characteristics had a significantly larger glycaemic response compared with the other clusters, with a mean difference of 0.7 mmol l^−1^ (95% CI 0.20–1.21; MARD-like versus SIRD- and MOD-like; *P* = 0.009; Supplementary Table 6 and Supplementary Fig. 5a). By contrast, in participants with BT2160 abundance below the median, there was no significant difference in glycaemic response between clusters (*P* = 0.6). This collectively suggests that the host pathophysiology (reflected by the clusters) and the gut microbiota interact and that the abundance of BT2160 in the gut flora influences the glycaemic response on top of the pathophysiology.

We also observed in a post hoc analysis that the 11 study participants who achieved remission of impaired fasting glucose after BSE treatment (fasting glucose below 6.1 mmol mol^−1^) had a higher abundance of BT2160 in the gut microbiota compared with those who remained in the prediabetic range (*P* = 0.03; *n* = 11 and 24, respectively; Extended Data Fig. 1 and Supplementary Fig. 5a). They had also lower plasma concentrations of GGT (a clinical marker coupled with hepatic fat content^2,8^; *P* = 0.01) and a tendency for lower HOMA-IR (Table 3 and Extended Data Fig. 1). Ten of them were in the cluster with MARD-like characteristics. In a regression model, we observed that the abundance of BT2160 in the gut microbiota and plasma concentration of GGT were independently and significantly associated with remission in response to BSE (*R*^2^ = 0.64 for the model using remission and non-remission as dependent variables and BT2160 abundance (*P* = 0.02) and plasma GGT (*P* = 0.01) as independent variables; Table 3 and Supplementary Fig. 5a), suggesting that these two variables could help identify those who are likely to achieve remission of impaired fasting glucose in response to BSE treatment.

**Extended Data Fig. 6:**
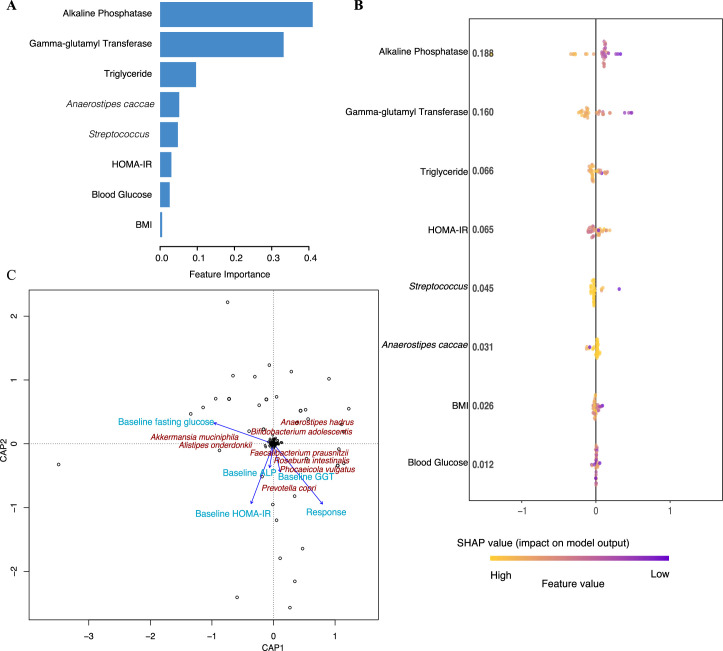
Relative importance of clinical and microbiota features in predicting the response to BSE. **a**, The relative importance of baseline variables (features) in predicting the change in fasting glucose after treatment with BSE. The measures of relative feature importance sum up to 1 and were generated by XGBoost. All participants receiving BSE were included in these analyses to investigate the importance of continuous baseline variables across all individuals. **b**, SHAP summary plot for the impact of baseline variables in predicting the change in fasting glucose in response to BSE treatment. Each point in the figure corresponds to a participant, and the SHAP value reflects the impact of the baseline variable in predicting the change in fasting glucose for that individual. For example, in the row corresponding to alkaline phosphatase, all individuals receiving BSE are plotted in accordance with how much baseline alkaline phosphatase predicts the change in fasting glucose. The panel is arranged based on the mean of the absolute SHAP values for each baseline variable. **c**, The impact of baseline clinical and microbiota variables on the glycemic response to BSE treatment using a distance‐based redundancy analysis model, calculated by the capscale function in R (the vegan package with the significance level based on the anova.cca function and PERMANOVA test with 10,000 permutations; P = 0.1). BMI is body mass index, blood glucose denotes fasting blood glucose, ALT is alkaline phosphatase, GGT is gamma‐glutamyl transferase, and HOMA‐IR is the homeostasis model assessment‐2 estimate of insulin resistance. Source data

Finally, to evaluate the combined importance of clinical variables and bacterial species to the variation in the response among all BSE recipients, considering also nonlinear effects, we used machine learning based on decision trees (Extreme Gradient Boosting (XGBoost)) and a distance-based redundancy analysis. The analyses showed that baseline alkaline phosphatase (ALP), GGT and triglycerides were associated with the glycaemic response to BSE (Extended Data Fig. 6a,b). Moreover, increased abundance of the health-associated bacteria *F. prausnitzii*, *Roseburia intestinalis*, *Phocaeicola vulgatus* and *Prevotella copri* was associated with the glycaemic response (Extended Data Fig. 6c). These data show that both hepatic markers and the baseline gut microbiota composition are associated with the response to BSE.

## Discussion

This trial shows that the response to sulforaphane-containing BSE in individuals with impaired fasting glucose differs based on the host pathophysiology and gut microbiota. In the full cohort, the 0.2 mmol l^−1^ reduction of fasting glucose in response to BSE compared with placebo did not meet the prespecified outcome of 0.3 mmol l^−1^. However, the data reveal marked variations in treatment response in individuals with MOD-like, SIRD-like and MARD-like characteristics, with an increased response in the cluster with MARD-like characteristics. The study also shows that the host pathophysiology (reflected by the clusters) and the abundance of the BT2160 transcriptional regulator in the gut microbiota interact and influence the glycaemic response to BSE. Taken together, this indicates a need to personalize interventions in prediabetes, considering that many compounds have moderate overall efficacy but considerable impact in certain subgroups, and these findings represent a step towards precision treatment of prediabetes based on the individual pathophysiology and gut microbiota.

Participants with a pronounced response to BSE had increased abundance of the butyrate producer *A. caccae* as well as sulphate-reducing *Desulfovibrio* sp., which support the growth of butyrate-producing bacteria^26^. Accordingly, butyrate kinase was elevated in the pronounced responders. The increased abundance of *Desulfovibrio* sp. and *A. caccae* may indicate a fermentative gut environment with higher butyrate production potential that could enhance the response to BSE. It is also possible that the sulphur component of sulforaphane might be used by *Desulfovibrio* to perform dissimilatory sulphate reduction and support butyrate producers^26^. Moreover, it is of interest that a recent study showed an association between elevated levels of *Desulfovibrio* and preserved beta-cell function following faecal microbiota transplantation^32^. Collectively, the gut microbiota composition of the pronounced responders is in line with their mild metabolic profile, characterized by reduced plasma concentration of clinical markers of NAFLD^33^ and a high proportion of individuals with MARD-like characteristics, with low BMI and low insulin resistance.

A particularly interesting difference between the pronounced responders and the remainder was the increased abundance in pronounced responders of the BT2160 operon that converts inactive glucosinolate to bioactive sulforaphane. Participants with high serum concentration of sulforaphane had also increased abundance of BT2160 in their gut microbiota. Moreover, the abundance of BT2160 was associated with improved glycaemic response in participants with MARD-like characteristics, with a significant interaction between the abundance of the operon and pathophysiological cluster.

While several stratification methods of potential relevance for diabetes have been proposed, the method used here has the advantage of including variables that can be obtained in clinical routine, as opposed to subgroups based on genetic risk variants or extensive clinical profiling^34^. Moreover, these clusters have been repeatedly demonstrated in diabetic cohorts of multi-ethnic origin^14–16^. Patient stratifications may, however, also be limited by the assumption of homogeneity within each cluster, the dependency on the background population and the potential change over time in response to treatment (which could make the approach more applicable to treatment-naive individuals with prediabetes as studied here). As the individual age and duration of hyperglycaemia will be higher in diabetes than in prediabetes, the disease progression and risk for complications may differ between prediabetes and diabetes clusters, despite pathophysiological similarities. We therefore used the clusters in this trial as a means to better understand the pathophysiological characteristics of those who benefit most from BSE.

MARD represents a mild form of diabetes, and future longitudinal studies will have to show what percentage of individuals with MARD-like characteristics and prediabetes develop overt diabetes. While it is likely that individuals with features of SIRD are more prone to severe disease progression, MARD is nevertheless the largest cluster of patients, representing 35–50% of all with diagnosed disease in various cohorts^14,16^. It is of interest that a 5-year longitudinal observational study showed that individuals with MARD had increased fatty liver index (which in turn was correlated with hepatocellular lipid content) and NAFLD fibrosis score over time^15^. This enhances the risk of disease deterioration and emphasizes the need for early intervention in this subgroup^15^. As beta-cell preservation is an important goal of diabetes prevention^4,5^, it is also of note that BSE significantly improved HOMA-B in individuals with MARD-like characteristics without any concomitant change in HOMA-IR.

Although no previous studies have investigated the differential treatment effect in prediabetic subgroups, it is of interest that patients with type 2 diabetes and MARD characteristics were reported to have lower glycaemic response to metformin compared with other subgroups^16^, highlighting the need for more tailored interventions with different therapeutic options. An observational study in prediabetes identified similar clusters and showed that the clusters with MOD-like and, particularly, SIRD-like characteristics have a larger fraction of individuals with combined impaired fasting glucose and impaired glucose tolerance (approximately 20–30%), in contrast to the cluster with MARD-like characteristics that had a higher proportion of individuals with isolated impaired fasting glucose^13^. This agrees with the pronounced glycaemic response in individuals with MARD-like characteristics to sulforaphane, which acts directly on the expression of gluconeogenic enzymes (in contrast to metformin) and offers a targeted means to interfere with exaggerated glucose production.

A meta-analysis that identified the association between fasting glycaemia and risk of future diabetes^35^ indicates that a reduction of fasting glucose from 6.4 to 6.1 mmol l^−1^, as observed in response to BSE, would correspond to a diminished hazard ratio for diabetes onset from ~12 to ~7, and a decrease from 6.4 to 6.0 mmol l^−1^, as observed in response to BSE in the cluster with MARD-like characteristics, corresponds to an ~50% reduction in hazard ratio. Although we cannot estimate the precise risk reduction based on these data, it is of relevance that BSE, despite not meeting the prespecified target of 0.3 mmol l^−1^, reduces fasting blood glucose by a similar magnitude (0.2 mmol l^−1^) to that observed with metformin (which decreased fasting blood glucose by ~0.2 mmol l^−1^ in the Diabetes Prevention Program and reduced diabetes incidence by 31% for 3 years)^36^. In view of the low cost of BSE, it is also of note that risk reductions as small as 5% have been shown to be clinically cost-effective owing to the large societal costs of diabetes^37^. Moreover, data from long-term studies on cancer prevention show that BSE has few adverse effects^11^. This is important in prediabetes, in which tolerance for side effects is presumably lower^38^. The provision of sulforaphane as a non-pharmaceutical food extract (BSE) rather than a traditional drug might also be attractive to individuals with impaired fasting glucose, who do not necessarily view themselves as being ill^39^.

The strengths of the study are the usage of a non-pharmaceutical plant-sourced compound as a treatment modality for prediabetes, the randomized placebo-controlled design, the investigation of the differential effect in pathophysiological subgroups of diabetes treatment-naive individuals and the analysis of microbiome profiles associated with the anti-hyperglycaemic response to BSE. The association between the abundance of the BT2160 transcriptional regulator in the gut microbiota and the glycaemic response to BSE (in addition to the individual pathophysiology) suggests a model for how the microbiota and host pathophysiology interact to influence treatment response that may have general implications for precision medicine.

The study also has a number of limitations. The follow-up time of 12 weeks does not allow for the analysis of long-term effects on glycaemic control, and future prospective studies will have to determine the rates of overt diabetes in the treatment groups. The CIs for the change of primary and secondary variables were not adjusted for multiple comparisons. Other study limitations are the discontinuation of 15 study participants with an overall higher frequency of gastro-intestinal side effects (without any systematic differences in baseline characteristics; Supplementary Table 1), who were not included in the full analysis set, and the self-selection process in recruitment, which could increase the risk that participants would be more motivated to perform lifestyle changes and be concordant with treatment than individuals with prediabetes in general. We observed no significant changes in dietary habits or physical activity between placebo and BSE during the study (Table 3), and the placebo-controlled randomized design makes it unlikely that the observed effects are merely the result of lifestyle changes in response to study participation.

In summary, the trial shows that the response to sulforaphane-containing BSE in individuals with impaired fasting glucose differs based on the individual pathophysiology and gut microbiota. In the full cohort, the effect of BSE did not reach the prespecified outcome of 0.3 mmol l^−1^ reduction of fasting glucose. The data show, however, that clustering of individuals with prediabetes into subgroups and analysing the abundance of the BT2160 operon in the gut microbiota can be used to identify those who benefit most from BSE. This opens an avenue for precision treatment of prediabetes based on the individual pathophysiology and gut microbiota composition that may have general implications. The mild side effect profile and the ability to provide sulforaphane-containing BSE, for example, as a ‘functional food’ could make it an attractive option for individuals with prediabetes and MARD characteristics, whereas other treatment modalities, including intensive lifestyle intervention programmes or drugs that specifically target high insulin resistance or fatty liver content, should be tested specifically in those with SIRD and MOD characteristics.

## Methods

### Trial design and oversight

The trial complies with all relevant ethical regulations, and the protocol was approved by the Regional Ethics Committee of Gothenburg (433-18). It started in December 2018 and was conducted as a randomized parallel-arm placebo-controlled double-blind trial in Gothenburg, Sweden (ClinicalTrials.gov NCT03763240) in accordance with the principles of the Declaration of Helsinki and Good Clinical Practice. The study was conducted at Gothia Forum, Sahlgrenska University Hospital, Gothenburg, Sweden, by academic investigators. Funders had no role in data interpretation. The trial was monitored by an independent monitor before, during and after its completion to ensure that it was carried out according to the protocol. All authors had access to the data, were involved in the writing and editing of the paper, vouch for the completeness and accuracy of the data, and agreed to submit the paper for publication.

### Participants

A random selection of members of the general population aged 35–75 years in Gothenburg, Sweden, and surrounding municipalities, who had registered addresses and Swedish personal numbers, received an invitation letter with study information and instructions on how to book a time for a screening visit. Gender was determined based on self-report and the official personal number. Participants received travel reimbursement but no other financial compensation.

### Inclusion criteria

Individuals were eligible to be included in the trial if all of the following criteria applied:

- Impaired fasting glucose, defined as fasting blood glucose at 6.1–6.9 mmol l^−1^
- Written informed consent
- Age 35–75 years; participating women of fertile age must have no current pregnancy, which was assessed by a pregnancy test
- BMI 27–45 kg m^−^^2^

### Exclusion criteria

- Diabetes mellitus based on previous documentation or treatment with anti-hyperglycaemic medication or diagnosed according to the World Health Organization criteria (random plasma glucose >11.1 mmol l^−1^ or fasting glucose >7.0 mmol l^−1^ or HbA1C ≥ 6.5%)
- Anti-diabetic medication
- Active liver disease
- At screening or at any subsequent visit, a level of aspartate aminotransferase (AST) or alanine aminotransferase (ALT) of more than three times the upper limit of the normal range
- Gastro-intestinal ailments that may interfere with the ability to adequately absorb sulforaphane
- At screening visit, creatinine >130 µmol l^−1^
- Coagulation disorder or current anti-coagulant therapy, which may be affected by BSE
- Diagnosed with a cardiovascular disease or known cardiovascular event, transient ischaemic attack, coronary by-pass surgery or other coronary vessel intervention within 6 months before enrolment
- Systemic glucocorticoid treatment
- Herbal treatment, defined as food supplement (except multivitamin treatment) with herbal or vegetable extracts that may affect blood glucose
- Participant unable to understand the study information
- Participation in another clinical trial, which may affect the outcome of the present study
- Any other physical or psychiatric condition or treatment that in the judgement of the investigator makes it difficult to participate in the study

### Trial procedures

All participants signed a written informed consent before study procedures were initiated. Participants were instructed not to conduct intense physical activity or drink alcohol 24 h before the study visits. They were also instructed to fast starting midnight and not use nicotine on the same day.

At the screening visit, the height and weight of each participant were measured and venous blood samples were drawn for analysis of glucose, creatinine, AST, ALT, GGT, ALP, bilirubin, prothrombin complex and thrombocytes. Blood samples were drawn at 7.30–10.00 a.m.

Individuals with fasting blood glucose between 6.1 and 6.9 mmol l^−1^ were invited to a second visit approximately 2 weeks later. At this visit, body weight was measured and fasting venous blood samples were drawn for analysis of primary, secondary and safety variables. At this baseline visit, stool samples were also collected.

Individuals who had fasting blood glucose between 6.1 and 6.9 mmol l^−1^ also at the second visit were randomized to receive BSE or placebo in a double-blind manner. The data from the second visit were used as baseline measures for analyses of primary and secondary variables. If blood glucose was 7.0 mmol l^−1^ or above, the individual was excluded and referred to primary healthcare.

The randomized participants were instructed to take BSE or placebo once daily in the morning. Concordance with treatment was noted in a diary and also checked at the final visit by counting the remaining doses. Study personnel contacted the participants by phone 2–4 weeks after the initiation of treatment to discuss concordance with treatment and side effects.

The third visit was scheduled on the same weekday as visit 2 (unless it was not possible because of public holidays) 12 weeks after the first dose of the study medication. At this visit, body weight was measured, stool samples were collected and fasting venous blood samples drawn for analysis of primary, secondary and safety variables.

Physical activity was assessed using the International Physical Activity Questionnaire (IPAQ), and dietary habits were assessed using items that had been validated in Swedish national health questionnaires^40^, which the participants completed during the second and third visits.

### Randomization

The randomization (in a 1:1 ratio between BSE and placebo) was generated by independent statisticians using a computer-based block randomization algorithm with balanced blocks. Allocation was concealed (via sealed envelopes) from the participants and study personnel until the end of the study. Thus, the generation of the random sequence, participant enrolment by study personnel and the allocation to randomization groups were clearly separated.

### Study compounds

BSE containing high amounts of the sulforaphane precursor glucoraphanin was provided by Lantmännen R&D. BSE is a dried powder of an aqueous extract of broccoli sprouts that provides a consistent and stable source of sulforaphane. The active formulation contained BSE with maltodextrin added as a bulking agent, whereas maltodextrin alone was used as placebo. The placebo looked, smelled and tasted similar to the active compound and had the same constituents except BSE. Study doses were provided as dry mixtures in sealed, non-transparent portion-size bags. Each BSE dose delivered 150 μmol of sulforaphane. Sulforaphane content was determined using reverse-phase high-performance liquid chromatography by Eurofins. No sulforaphane was detected in the placebo. The mixtures of BSE and placebo were suspended with approximately 1 dl water and ingested orally once daily in the morning.

Safety studies of BSE in healthy volunteers have revealed no evidence of systematic, clinically significant adverse effects^11,41,42^. This has been confirmed in several clinical trials with healthy volunteers as well as, for example, patients with recurrent prostate cancer, where doses of up to 400 μmol sulforaphane have been used^37,42,43^. The most commonly reported side effects are indigestion, belching or loose stools^12,41–43^.

### Outcomes

Venous blood samples were taken between 7.30 and 10.00 in the morning. Fasting blood glucose from venous samples was measured at the study centre using a HemoCue Glucose System (HemoCue AB). All other blood analyses were performed at the central hospital laboratory (Gothenburg, Sweden). Homeostasis model assessment-2 estimates of insulin resistance (HOMA-IR) and beta-cell function (HOMA-B) were determined as previously described^44^.

The primary variable was fasting blood glucose, and the primary objective was to test the hypothesis that BSE improves fasting blood glucose using intraindividual comparisons before (baseline) and after treatment in the BSE group relative to the placebo group. The secondary variables were the change from baseline in HbA1c, BMI, insulin resistance (measured by HOMA-IR), insulin secretion (measured by HOMA-B), fasting blood lipids and a fatty liver index based on BMI, waist circumference, triglycerides and GGT^45^. Liver parameters, including GGT, ALP, AST, ALT and bilirubin, were also measured, and haemoglobin, thrombocytes, thyroid-stimulating hormone, creatinine and estimated glomerular filtration rate (based on creatinine) were analysed as safety variables. Insulin clearance was estimated using the fasting C-peptide-to-insulin ratio.

### Patient-reported outcomes

Participants completed the IPAQ, which assesses intense and moderate physical activity as well as walking during the past 7 days (ref. ^46^). The questionnaire was completed at baseline and at the last visit by participants. Responses were converted to metabolic equivalent task minutes per week according to the IPAQ scoring protocol^46^.

Dietary habits were assessed using a food frequency questionnaire previously used in public health surveys. The self-reported frequency of intake of vegetables, lentils and root vegetables; fruit and berries; fish and shellfish; sausages; chocolate and sweets; cakes, buns and cookies; cheese; and sugared beverages was recorded and scored according to a reference indicator from the National Food Administration^41^. The items were summed to a total score from 0 to 9 (with 9 indicating a diet most adherent to official food recommendations).

### Clustering of study participants

The data-driven clustering based on diabetes-relevant traits was conceived in the All New Diabetics In Scania (ANDIS) cohort^14^. ANDIS aims to register all incident cases of diabetes in Scania, which is one of the largest regions in Sweden with 1,200,000 inhabitants. Over 27,000 diabetic patients (>90% of the estimated number of eligible cases in the region) are included. The clustering is based on continuous measures of BMI, age, fasting glucose, C-peptide and HbA1c as well as the presence or absence of glutamic acid decarboxylase antibodies (GADA) as a binary variable. The method, which is described in detail in ref. ^14^, is based on *k*-means clustering and has highlighted five clusters of patients with diabetes, each with different pathophysiological characteristics^14,15^.

The alignment of study participants with the clusters was performed using the baseline data of each participant. GADA was not measured in this study, but all participants were assumed to have non-autoimmune diabetes based on disease history (type 1 diabetes was an exclusion criterion in the study). The clustering was based on bootstrapping. In every round, the 8,980 individuals used to analyse the original clusters in ref. ^14^ were sub-sampled, such that 60% of the cohort was randomly selected and clustered. The centroid, represented by the relative coordinates of the included variables, was determined for each cluster. The study participants were then assigned to one of the clusters based on the nearest Euclidean distance to the cluster centroids. This procedure was repeated in a bootstrapping algorithm, and the number of counts for the different cluster was summed for each study participant. The fraction of repeats that a study participant was assigned to the same cluster was used to determine a cluster alignment score from 0 to 1. A score of 1 means that the participant was assigned to the same cluster in every repeat.

As age at diagnosis of diabetes is used to cluster diabetes patients^14^, while age at diagnosis of prediabetes (that is, age at study inclusion) was used in this trial, disease progression and risk for complications may differ, despite pathophysiological similarities between clusters. Thus, the rationale for using the clusters was not to predict complications but to examine the glycaemic response in individuals with different clinical and pathophysiological characteristics.

### Replication cohort

The clustering was also performed using baseline data in a separate cohort of individuals taking part in a longitudinal study to examine the influence of lifestyle on diabetes progression (ClinicalTrials.gov NCT05006508). The study complies with all relevant ethical regulations, and the protocol was approved by the Swedish Ethics Review Authority 2021-06830-01. Study participants were recruited by advertisements in 2021–2024. Individuals above 35 years of age across Sweden were eligible to participate after giving informed consent. Those who had not been diagnosed with type 1, type 2 or secondary diabetes completed a diabetes risk assessment questionnaire (the Finnish Diabetes Risk Score questionnaire, ranging from 0 to 26 with higher scores corresponding to higher diabetes risk) at baseline. Participants with a Finnish Diabetes Risk Score at 15 or above were requested to leave blood samples for analysis of fasting glucose, C-peptide (to determine HOMA-B and HOMA-IR) and HbA1c to study their metabolic profile and better understand which factors contribute to the progression of diabetes over time. Those who were 35–75 years old with a fasting blood glucose between 6.1 and 6.9 mmol l^−1^ at baseline and BMI 27–45 kg m^−^^2^ (corresponding to the study criteria of the BSE trial) were clustered with the same methodology used for the participants of the BSE trial.

### DNA extraction, library preparation and shotgun metagenomic sequencing of faecal samples

All study participants collected their own faecal samples at room temperature before visit 2 and 3. The faecal samples were delivered on the same day of sampling to the study centre, where they were stored at −80 °C. To use the samples to the largest extent possible, they were analysed even for participants who had non-complete clinical follow-up data. Total genomic DNA was isolated from 100–150 mg of faecal material using a modification of the International Human Microbiome Standards DNA extraction protocol Q7 (ref. ^47^). Samples were extracted in Lysing Matrix E tubes (MP Biomedicals) containing ASL buffer (Qiagen), vortexed for 2 min and lysed by two cycles of heating at 90 °C for 10 min followed by two bursts of bead beating at 5.5 m s^−1^ for 60 s in a FastPrep-24 Instrument (MP Biomedicals). After each bead-beating burst, samples were placed on ice for 5 min. Supernatants were collected after each cycle by centrifugation at 4 °C. Supernatants from the two centrifugation steps were pooled, and a 600 µl aliquot from each sample was purified using the QIAamp DNA Mini kit (Qiagen) in the QIAcube (Qiagen) instrument using the procedure for human DNA analysis. Samples were eluted in 200 µl of AE buffer (10 mM Tris·Cl; 0.5 mM EDTA; pH 9.0). Libraries for shotgun metagenomic sequencing were prepared by a PCR-free method; library preparation and sequencing were performed at Novogene on a NovaSeq instrument (Illumina) with 150 bp paired-end reads and at least 6 G data per sample.

### Faecal metagenomic profiling and bioinformatic analysis

The metagenomic reads were quality filtered and trimmed using fastq_quality_trimmer from the fastX toolkit (https://github.com/lianos/fastx-toolkit/). To remove human contamination, reads were mapped against the human genome (hg19) using Bowtie2 v2.4.4 (ref. ^48^). Filtered reads passing the quality criteria were then mapped using Kraken2 with default settings against the RefSeq database (release 107). Abundance estimation was performed using Bracken for all reads with a minimum read length of 100 bp. Gene count estimation was performed on a previously published gene catalogue containing 15,186,403 non-redundant microbial genes^17^. Kyoto Encyclopedia of Genes and Genomes ontology annotations were then performed for microbial functional profiling based on MEDUSA^49^. Butyrate kinase gene (*buk*) representing one of the bacterial butyrate-producing pathways^27^ was profiled using hidden Markov models to screen the gene catalogue and to identify the butyrate producers among the metagenomic species by HMMER^50^.

The BT2156–BT2160 protein sequences were downloaded from RefSeq (WP_008763945- WP_00876394) and mapped against *Bacteroides* D2 (accession id NZ_CP102261) and *Bacteroides* DM10 (accession id CP060488) based on the reference genomes of the species used in the RefSeq database (release 107). The gene count estimation of BT2160, the transcriptional regulator of the operon, was performed on the gene catalogue of the non-redundant microbial genes detailed above, and statistical significance was determined based on the proportion of permutation test statistics greater than or equal to the observed statistic (using 10,000 permutations with a random shuffle function in R 4.1.0).

PCo analysis was performed on Bray–Curtis dissimilarity at the species level, calculated based on species abundances, and significance was determined by PERMANOVA test using the adonis2 function with 10,000 permutations. Significantly differential abundant species tables were obtained using the deseq2 package with adjustment for subjects at different visits. The *P* value adjustment for significantly altered taxa was performed by the default setting in deseq2 using the false discovery rate according to the Benjamini–Hochberg method. Correlation of gut microbiota species abundances with clinical parameters was performed using distance-based redundancy analysis with the capscale function using anova.cca and 10,000 permutations. The functions used in these analyses are implemented in the vegan package (Community Ecology Package-R package version 1.17-8). All statistical analyses involving faecal whole-genome metagenomics were performed in R 4.1.0.

### Continuous baseline variables that predict response to BSE

We used XGBoost, an ensemble machine learning technique based on decision trees, to identify continuous baseline variables that predict the change in fasting glucose after treatment of the study drugs. The method develops a multivariable ensemble of prediction models that were used to identify the strongest predictors of response. The optimal values for hyperparameters for each outcome were detected by performing a grid search on several possible combinations of different variables. The hyperparameters include the number of trees, learning rate, minimal loss to expand on a leaf node, maximum tree depth and subsample proportion. All other parameters were used at their default values. The package XGBoost version 1.6.0.1 was used in R 4.1.0.

We computed the relative importance of each variable predicting the outcome using *F* scores in XGboost, calculated as the sum of Gini improvement among the corresponding splits within a tree averaged over all the trees. In addition, we implemented Shapley Additive Explanations (SHAP), for easy interpretation of the machine learning model output. The SHAP value in this analysis is the mean absolute individual feature-level impact on the model. The training set in our models consisted of a randomly selected subset of 80% of the study participants, and the testing set was composed of the remaining 20%. The model was based on data from the training set; the testing set was independent of the training process and was used only for performance evaluation after the model was established.

### Measurement of sulforaphane in serum

The concentration of sulforaphane in serum samples from participants was measured as previously described^51^. The methodology is based on analysing dithiocarbamate levels in patient serum by the cyclocondensation reaction for measurement of sulforaphane and its metabolites. Absence of sulforaphane in samples from the placebo group was verified by parallel measurements of the sulforaphane concentration in serum from placebo-treated participants. The difference in the average abundance of BT2160 in baseline and post-treatment samples between participants with low and high sulforaphane concentration in serum was compared using a weighted least squares analysis, adjusted for body surface area.

### Statistical analysis

The primary endpoint was the intraindividual change in fasting glucose from baseline in response to BSE or placebo, which was analysed using a linear model adjusted for BMI and variation in HOMA-IR. The comparison of fasting glucose was also complemented with an ANCOVA model. Secondary endpoints included the intraindividual change in secondary variables from baseline in response to BSE or placebo and were analysed using a linear model as for the primary endpoint. Normality was verified for the major clinical variables using normal probability plots.

The full analysis set includes all participants who have clinical measures after randomization, independent of concordance with treatment.

In view of observations in patients with type 2 diabetes that serum triglyceride concentration is associated with the response to BSE, individuals below or above the median serum triglyceride concentration were also analysed separately.

The data-driven clustering method was published after the design of this study, and the investigation of clusters is a post hoc analysis. The primary and secondary variables were compared between BSE and placebo within each cluster of participants using corresponding linear models as applied to the full cohort. The interaction between treatment and subgroup was analysed by a linear model with one term for treatment (BSE or placebo), one term for the subgroup and an interaction term for the treatment and subgroup. Baseline variables were compared between the three clusters using ANOVA followed by Bonferroni corrections to obtain an overall *P* value for the variation between all three groups and for pairwise comparisons between groups.

The interaction between the abundance of BT2160 (log values) and pathophysiological subgroup (MARD versus MOD and SIRD) was analysed by a linear model with one term for BT2160 abundance, one term for subgroup and an interaction term for BT2160 and subgroup with the change of fasting glucose in response to BSE as the dependent variable. The analysis was adjusted for variation in body surface area between participants using the standard Du Bois formula.

The study was designed to have 80% power to detect a treatment effect of 0.3 mmol l^−1^ between BSE and placebo. The standard deviation of change in fasting blood glucose over 12 weeks is 0.63 mmol l^−1^, based on analyses in our longitudinal cohorts of subjects with impaired fasting blood glucose. At alpha 0.05, at least 74 study participants were needed.

Two-sided *P* values of 0.05 or less were considered to indicate statistical significance. Summary statistics are generally presented as point estimates with 95% CI unadjusted for multiple comparisons. Statistical analyses were performed using SPSS (version 26, IBM) or R 4.1.0.

### Reporting summary

Further information on research design is available in the Nature Portfolio Reporting Summary linked to this article.

## Supplementary information

Supplementary Information Supplementary Tables 1–6, Figs. 1–8 and notes.

Reporting Summary

Supplementary Data 1 Study protocol and statistical analysis plan.

Supplementary Data 2 Statistical source data for supplementary tables and figures.

## Source data

Source Data Fig. 1 Statistical source data for Fig. 1.

Source Data Fig. 2 Statistical source data for Fig. 2 with (a) cluster designation in the study cohort, (b) cluster designation in the replication cohort, (c) principal coordinates of Bray–Curtis dissimilarities and (d,e) altered taxa.

Source Data Fig. 3 Statistical source data for Fig. 3 with principal coordinates of Bray–Curtis dissimilarities for participants with a pronounced and less pronounced response, respectively, and significantly altered taxa.

Source Data Table 1 Statistical source data for Table 1 with baseline variables.

Source Data Table 2 Statistical source data for Table 2 with reported adverse events.

Source Data Table 3 Statistical source data for Table 3 with outcome variables at baseline and after treatment.

Source Data Extended Data Fig. 1 Source data for Extended Data Fig. 1 with outcome variables before and after treatment in all participants.

Source Data Extended Data Fig. 2 Source data for Extended Data Fig. 2 with outcome variables before and after treatment in all participants.

Source Data Extended Data Fig. 3 Source data for Extended Data Fig. 3 with outcome variables before and after treatment in participants with MARD-like characteristics.

Source Data Extended Data Fig. 4 Source data for Extended Data Fig. 4 with outcome variables before and after treatment in participants with MARD-like characteristics.

Source Data Extended Data Fig. 5 Source data for Extended Data Fig. 5 with principal coordinates at baseline and after treatment.

Source Data Extended Data Fig. 6 Source data for Extended Data Fig. 6 with baseline variables used to predict the change in fasting glucose in response to BSE.

## Data Availability

Raw metagenomic sequence data have been deposited in the European Molecular Biology Laboratory-European Bioinformatics Institute European Nucleotide Archive under accession number PRJEB77105. To remove human contamination, reads were mapped against the human genome (hg19) using Bowtie2 v2.4.4. Filtered reads passing the quality criteria were then mapped using Kraken2 with default settings against the RefSeq database (release 107). The BT2156–BT2160 protein sequences were downloaded from RefSeq (WP_008763945–WP_00876394) and mapped against *Bacteroides* D2 (accession id NZ_CP102261) and *Bacteroides* DM10 (accession id CP060488) based on the reference genomes of the species used in the RefSeq database (release 107). All clinical data supporting the findings of this study and the study protocol are available in the Article and Supplementary Information. Source data are provided with this paper. All other data that support the findings of this study are available from the corresponding author upon reasonable request. De-identified individual and/or study-level data will be shared with researchers who provide a methodologically sound proposal and if regulatory criteria are met. Access to anonymized data may be granted following review (time frame <20 office days) to ensure compliance with relevant ethical and legal considerations.
