# Supplementary material for: Effect of broccoli sprout extract and baseline gut microbiota on fasting blood glucose in prediabetes: a randomized, placebo-controlled trial
Source: Nat Microbiol. 2025 Feb 10;10(3):681–93. doi: 10.1038/s41564-025-01932-w (PMC11879859; doi:10.1038/s41564-025-01932-w)
Supplement: Supplementary file 1 — Supplementary Tables 1–6, Figs. 1–8 and notes. [file 41564_2025_1932_MOESM1_ESM.pdf]

nature microbiology

Supplementary information

https://doi.org/10.1038/s41564-025-01932-w

Effect of broccoli sprout extract and baseline gut microbiota on fasting blood glucose in prediabetes: a randomized, placebo-controlled trial

In the format provided by the authors and unedited

Supplementary Notes

Cluster analysis of study participants

There were marked differences in baseline variables between clusters (Supplementary Table 4). Participants in the MARD-like cluster had significantly lower BMI, HOMA-IR, HOMA-B, fasting insulin and C-peptide, fatty liver index and triglyceride concentration as well as higher fasting C-peptide to insulin ratio (an estimate of insulin clearance) compared with the other clusters. Individuals in the MOD-like cluster had considerably lower age compared with the other two clusters, while the estimated glomerular filtration rate (eGFR) was lower in SIRD-like and MARD-like compared with MOD-like individuals. The eGFR was still in normal range, but it is of note that SIRD and MARD are the two clusters with the highest incidence of chronic kidney disease in prospective analyses of patients with diagnosed diabetes[1]. The variation of mean age, BMI, HOMA-IR and HOMA-B between clusters was similar to that observed in large cohorts of patients with diagnosed diabetes[2,3]. Fasting blood glucose and HbA1c at baseline did not differ between clusters, which is also in agreement with observations in newly diagnosed patients (although their absolute values are naturally higher than in the study participants).

The larger effect of BSE in participants with MARD-like characteristics is of interest in light of the association between serum triglyceride concentration and the change of fasting glucose in response to BSE that was observed in patients with type 2 diabetes[3]. Low concentration of serum triglycerides is one of the distinctive features of MARD, and the association between this phenotype and greater treatment response is further emphasized by observations that study participants with baseline triglyceride concentration below median (1.3 mmol/l) had a larger reduction of fasting glucose in response to BSE (0.3 mmol/l mean difference versus placebo; 95% CI: -0.6 to -0.1; n=35, of whom 27 had MARD characteristics) compared with participants with triglyceride concentration above median (for whom the mean difference of fasting glucose between BSE and placebo was 0.1 mmol/l; 95% CI: -0.4 to 0.3).

Cluster analysis in replication cohort

Since this is the first extension of the data-driven clustering to prediabetes, we examined the robustness of cluster assignment by determining the distance of each participant to the cluster centroid and calculating a cluster alignment score (with 1 indicating high correspondence of the individual traits with the average cluster characteristics). The mean score of the participants was 0.94, demonstrating an overall good alignment within the clusters. To further assess the stability and reproducibility of the cluster distribution in prediabetes, we utilized data from an independent cohort of 164 individuals with impaired fasting blood glucose (Supplementary Table 5). Of those, 12.8% were SIRD-like, 28.7% MOD-like and 58.5% MARD-like, as compared with 21.3%, 21.3% and 57.3%, respectively, in the present study (Fig. 1a,b). This indicates that the MARD-like cluster, in particular, had a stable distribution across cohorts.

Supplementary Tables

Supplementary Table 1. **Demographic and baseline characteristics of participants in the full analysis set and participants who discontinued.** [a]

Characteristic	Full analysis set (n=74)	Discontinued (n=15)	All (n=89)
**Age – yr**	63±9	61±11	63±9
**Male – no (%)**	29 (66)	9 (60)	57 (64)
**Fasting glucose – mmol/l**	6.4±0.2	6.4±0.1	6.4±0.2
**HbA1c – mmol/mol**	38.2±3.9	38.1±5.0	38.2±4.1
**Body mass index**[b]	31.7±3.3	32.4±5.2	32.1±3.8
**HOMA-B**	117.6±28.1	123.5±33.3	118.6±28.9
**HOMA-IR**	5.3±3.1	6.2±4.1	5.4±3.3
**Fasting insulin – mIE/l**	18.4±10.5	21.5±13.7	18.9±11.1
**Fasting C-peptide – nmol/l**	1.20±0.39	1.29±0.55	1.22±0.42
**Fasting C-peptide/insulin ratio**	0.075±0.021	0.067±0.016	0.074±0.021
**Bilirubin – μmol/l**	9.9±4.3	10.0±4.3	9.9±4.3
**ALP – μkat/l**	1.2±0.3	1.0±0.3	1.1±0.3
**GGT – μkat/l**	0.6±0.5	0.8±0.6	0.7±0.5
**AST – μkat/l**	0.5±0.2	0.5±0.1	0.4±0.2
**ALT – μkat/l**	0.6±0.3	0.6±0.2	0.6±0.3
**Fatty liver index**[c]	75.1±18.8	83.2±18.4	76.4±18.9
**Total cholesterol – mmol/l**	5.1±1.0	5.2±1.2	5.1±1.0
**LDL – mmol/l**	3.5±1.0	3.5±1.1	3.5±1.0
**HDL – mmol/l**	1.4±0.4	1.5±0.5	1.4±0.4
**Triglycerides – mmol/l**	1.4±0.6	1.5±0.7	1.5±0.6
**Creatinine – μmol/l**	78.7±15.4	77.3±12.3	78.5±14.9
**Estimated glomerular filtration rate – ml/min/1.73 m^2**	74.9±11.3	76.5±10.4	75.1±11.1

[a] Plus–minus values are means ± SD. HbA1c denotes glycated hemoglobin, ALP alkaline phosphatase, GGT glutamyl transferase, AST aspartate aminotransferase, ALT alanine aminotransferase. LDL and HDL denote low- and high-density lipoprotein, respectively. HOMA-IR and HOMA2-B denote homeostasis model assessment-2 estimates of insulin resistance and beta-cell function, respectively.
[b] The body-mass index (BMI) is the weight in kilograms divided by the square of the height in meters.
[c] The fatty liver index was calculated based on BMI, waist circumference, triglycerides and GGT.

Supplementary Table 2. **Fasting glucose at baseline and after treatment.** [a]

Primary variable	Average in all participants	Average in SIRD-like	Average in MOD-like	Average in MARD-like
**Fasting glucose at baseline in placebo group – mmol/l**	6.4±0.2	6.4±0.2	6.4±0.2	6.4±0.2
**Fasting glucose at baseline in BSE group – mmol/l**	6.4±0.2	6.4±0.1	6.5±0.1	6.4±0.2
**Fasting glucose after treatment in placebo group – mmol/l**	6.2±0.5	6.4±0.5	5.8±0.6	6.3±0.4
**Fasting glucose after treatment in BSE group – mmol/l**	6.1±0.5	6.4±0.3	6.3±0.3	6.0±0.6
**Intraindividual change of fasting glucose after treatment compared with baseline in placebo group – mmol/l**	-0.2±0.6	0.0±0.4	-0.6±0.7	-0.1±0.5
**Intraindividual change of fasting glucose after treatment compared with baseline in BSE group – mmol/l**	-0.3±0.5	0.0±0.3	-0.2±0.1	-0.4±0.5

[a] Primary outcome variable at baseline and after 12 weeks of treatment and the intraindividual change in participants assigned to placebo or BSE. Plus–minus values are means ± SD in all participants (n=39 placebo and 35 BSE) as well as in participants with SIRD-like (n=9 placebo and 8 BSE), MOD-like (n=10 placebo and 3 BSE) and MARD-like (n=20 placebo and 24 BSE), respectively. The number of participants and the corresponding mean values refer to those who had measures of fasting glucose both before and after treatment.

Supplementary Table 3. **Baseline characteristics of participants in different clusters.**[a]

**Characteristic**	SIRD-like (n=19)	MOD-like (n=19)	MARD-like (n=51)	Overall P value[c]	P value[d] SIRD vs. MOD	P value[d] MARD vs. SIRD	P value[d] MARD vs. MOD
**Age – yr**	65±6	51±8	67±6	0.4	1E-8	0.6	6E-14
**Male – no (%)**	14 (74)	10 (53)	33 (65)	1E-13	0.5	1.0	1.0
**Fasting glucose – mmol/l**	6.4±0.2	6.4±0.2	6.4±0.2	0.7	1.0	1.0	1.0
**HbA1c – mmol/mol**	38.2±3.4	38.4±6.1	38.1±3.5	1.0	1.0	1.0	1.0
**Body mass index**	33.1±3.5	36.3±4.2	30.2±2.0	8E-11	0.003	0.002	6E-11
**HOMA-B**	154±22	124±25	103±19	6E-13	0.0001	3E-13	0.001
**HOMA-IR**	8.6±3.3	6.9±3.6	3.7±1.5	1E-10	0.1	6E-10	0.00003
**Fasting insulin – mIE/l**	29.9±10.9	23.7±12.4	12.9±5.1	7E-11	0.09	3E-10	3E-5
**Fasting C-peptide – nmol/l**	1.71±0.35	1.32±0.38	0.99±0.25	4E-13	0.0005	3E-13	0.0003
**Fasting C-peptide/insulin ratio**	0.061±0.015	0.062±0.015	0.083±0.020	2E-6	1.0	0.001	8E-5
**Bilirubin – μmol/l**	10.5±4.8	7.6±2.6	10.5±4.4	0.03	0.1	1.0	0.03
**ALP – μkat/l**	1.1±0.3	1.2±0.3	1.1±0.3	0.7	1.0	1.0	1.0
**GGT – μkat/l**	0.9±0.6	0.6±0.3	0.6±0.5	0.05	0.3	0.04	1.0
**AST – μkat/l**	0.6±0.2	0.4±0.1	0.4±0.2	0.01	0.03	0.02	1.0
**ALT – μkat/l**	0.7±0.3	0.5±0.2	0.5±0.3	0.07	0.3	0.07	1.0
**Fatty liver index**[b]	83.4±22.8	86.4±18.1	70.2±15.1	0.001	1.0	0.02	0.003
**Total cholesterol – mmol/l**	4.8±1.0	5.0±1.2	5.2±1.0	0.4	1.0	0.6	1.0
**LDL – mmol/l**	3.4±1.0	3.4±1.2	3.6±0.9	0.6	1.0	1.0	1.0
**HDL – mmol/l**	1.2±0.3	1.2±0.2	1.6±0.4	0.00001	1.0	0.0003	0.0004
**Triglycerides – mmol/l**	1.8±0.6	1.7±0.8	1.2±0.4	0.0001	1.0	0.0003	0.02
**Creatinine – μmol/l**	86.4±13.7	72.7±10.4	77.6±15.7	0.01	0.01	0.08	0.6
**eGFR – ml/min/1.73 m²**	69.3±8.5	85.6±10.6	73.4±9.7	2E-6	4E-6	0.4	0.00003

[a] Plus–minus values are means ± SD. HbA1c denotes glycated hemoglobin. HOMA-IR and HOMA2-B denote homeostasis model assessment-2 estimates of insulin resistance and beta-cell function, respectively. ALP is alkaline phosphatase, GGT glutamyl transferase, AST aspartate aminotransferase,

ALT alanine aminotransferase, LDL and HDL denote low- and high-density lipoprotein, respectively, and eGFR is estimated glomerular filtration rate.

[b] The fatty liver index was calculated based on BMI, waist circumference, triglycerides and GT.

[c] Overall P value with two-sided testing in ANOVA models including all three clusters.

[d] Bonferroni-corrected P values of direct comparisons between SIRD-like and MOD-like, MARD-like and MOD-like and MARD-like and SIRD-like, respectively, using two-sided testing.

Supplementary Table 4. **Demographic and baseline characteristics of participants in the replication cohort and the study cohort, respectively.** [a]

Characteristic	Replication cohort (n=164)	Study cohort (n=89)
**Age – yr**	62±7	63±9
**Male – no (%)**	39 (24)	57 (64)
**Fasting glucose – mmol/l**	6.4±0.2	6.4±0.2
**HbA1c – mmol/mol**	39.3±3.7	38.2±4.1
**Body mass index**[b]	32.4±4.0	32.1±3.8
**HOMA-B**	111±29	119±29
**HOMA-IR**	2.6±1.0	5.4±3.3
**Fasting C-peptide – nmol/l**	1.10±0.46	1.22±0.42
**ALP – μkat/l**	1.2±0.4	1.1±0.3
**GGT – μkat/l**	0.5±0.3	0.7±0.5
**AST – μkat/l**	0.5±0.2	0.4±0.2
**ALT – μkat/l**	0.6±0.5	0.6±0.3
**Total cholesterol – mmol/l**	5.3±1.3	5.1±1.0
**LDL – mmol/l**	3.4±1.3	3.5±1.0
**HDL – mmol/l**	1.4±0.3	1.4±0.4
**Triglycerides – mmol/l**	1.6±0.7	1.5±0.6
**Creatinine – μmol/l**	68.6±12.9	78.5±14.9
**Estimated glomerular filtration rate – ml/min/1.73 m^2**	77.7±11.6	75.1±11.1
**Baseline physical activity – metabolic minutes per week**[c]	2334±2681	2317±1704

[a] Data from an independent cohort of individuals with impaired fasting blood glucose (n=164; Methods) and the study cohort. Plus–minus values are means ± SD. HbA1c denotes glycated hemoglobin, ALP alkaline phosphatase, GGT glutamyl transferase, AST aspartate aminotransferase, ALT alanine aminotransferase.

[b] The body-mass index (BMI) is the weight in kilograms divided by the square of the height in meters.

[c] Self-reported data via the International Physical Activity Questionnaire.

Supplementary Table 5. **Baseline characteristics of participants in different clusters in the replication cohort.[a]**

Characteristic	SIRD-like (n=21)	MOD - like (n=47)	MARD-like (n=96)	All (n=164)	Overall P value[b]	P value[c] SIRD vs. MOD	P value[c] MARD vs. SIRD	P value[c] MARD vs. MOD
**Age – yr**	65±7	55±6	65±6	62±7	9E-12	6E-8	1.0	2E-14
**Male – no (%)**	10 (48)	4 (9)	25 (26)	39 (24)	0.001	0.001	0.09	0.054
**Fasting glucose – mmol/l**	6.4±0.2	6.4±0.2	6.4±0.2	6.4±0.2	0.7	1.0	1.0	1.0
**HbA1c – mmol/mol**	39.1±5.3	39.7±3.9	39.1±3.2	39.3±3.7	0.4	1.0	1.0	1.0
**Body mass index**	36.0±4.3	35.3±3.3	30.1±2.5	32.4±4.0	3E-21	1.0	4E-13	3E-17
**HOMA-B**	161±30	114±19	99±20	111±29	5E-24	3E-14	2E-19	0.0002
**HOMA-IR**	4.3±1.1	2.7±0.7	2.2±0.6	2.6±1.0	6E-23	4E-14	2E-23	0.001
**Fasting C-peptide – nmol/l**	1.57±0.21	1.29±0.27	0.96±0.19	1.10±0.46	6E-8	0.03	0.09	0.0004
**ALP – μkat/l**	1.1±0.3	1.2±0.3	1.1±0.5	1.2±0.4	0.5	1.0	1.0	0.8
**GGT – μkat/l**	0.5±0.2	0.6±0.3	0.5±0.3	0.5±0.3	0.6	1.0	1.0	0.9
**AST – μkat/l**	0.6±0.2	0.6±0.3	0.5±0.2	0.5±0.2	0.7	1.0	1.0	1.0
**ALT – μkat/l**	0.7±0.2	0.7±0.6	0.5±0.3	0.6±0.5	0.2	1.0	0.9	0.3
**Total cholesterol – mmol/l**	5.0±1.5	5.3±1.1	5.3±1.3	5.3±1.3	0.5	0.9	1.0	1.0
**LDL – mmol/l**	3.4±1.5	3.4±1.0	3.3±1.3	3.4±1.3	0.9	1.0	1.0	1.0
**HDL – mmol/l**	1.3±0.2	1.4±0.4	1.5±0.5	1.4±0.3	0.1	1.0	0.1	0.6
**Triglycerides – mmol/l**	1.7±0.9	1.8±0.8	1.5±0.7	1.6±0.7	0.05	1.0	0.6	0.06
**Creatinine – μmol/l**	74.5±11.3	65.7±11.5	69.4±13.9	68.6±12.9	0.3	0.4	1.0	1.0
**Estimated glomerular filtration rate – ml/min/1.73 m²**	76.3±10.5	81.5±13.5	72.9±16.8	77.7±11.6	0.2	1.0	1.0	0.2

[a] Cluster distribution was evaluated in an independent cohort of individuals with impaired fasting blood glucose (n=164; Methods). Plus–minus values are means ± SD. HbA1c denotes glycated hemoglobin. HOMA-IR and HOMA2-B denote homeostasis model assessment-2 estimates of insulin resistance and beta-cell function, respectively.
[b] Overall P value with two-sided testing from ANOVA models including all three clusters.
[c] Bonferroni-corrected P values of direct comparisons between SIRD-like and MOD-like, MARD-like and MOD-like and MARD-like and SIRD-like, respectively, using two-sided testing.

Supplementary Table 6. **Effect of BSE on primary and secondary endpoints in different clusters.** [a]

Endpoint	Mean difference in SIRD-like (95% CI)[b]	Mean difference in MOD-like (95% CI)[b]	Mean difference in MARD-like (95% CI)[b]	Mean difference in all (95% CI)[b]
**Change in fasting glucose – mmol/l**	-0.05 (-0.37 to 0.27)	0.31 (-0.67 to 1.19)	-0.38 (-0.64 to -0.12)	-0.2 (-0.44 to -0.01)
**Change in HbA1c – mmol/mol**	-0.9 (-2.7 to 0.9)	-1.6 (-3.0 to -0.2)	0.0 (-1.4 to 1.5)	-0.3 (-1.3 to 0.6)
**Change in body mass index[c]**	-0.4 (-1.4 to 0.7)	-1.0 (-2.3 to 0.2)	-0.4 (-1.0 to 0.2)	-0.4 (-0.8 to 0.0)
**Change in HOMA2-B**	-7.4 (-29 to 14)	-11.5 (-47 to 24)	14.7 (3.3 to 26)	7.1 (-3.2 to 17.4)
**Change in HOMA2-IR**	1.2 (-1.8 to 4.2)	1.5 (-1.3 to 4.3)	0.3 (-0.4 to 1.1)	0.9 (-0.1 to 1.9)
**Change in fasting insulin – mIE/l**	4.3 (-6.0 to 14.5)	4.2 (-11.3 to 19.8)	1.4 (-1.0 to 3.9)	3.4 (0.1 to 6.8)
**Change in fasting C-peptide – nmol/l**	0.02 (-0.26 to 0.29)	0.11 (-0.12 to 0.36)	0.06 (-0.07 to 0.16)	0.07 (-0.02 to 0.17)
**Change in fasting C-peptide/insulin ratio**	-0.007 (-0.020 to 0.006)	-0.001 (-0.026 to 0.024)	-0.004 (-0.020 to 0.013)	-0.006 (-0.016 to 0.005)
**Change in fatty liver index[d]**	-1.6 (-5.4 to 2.3)	-2.7 (-10.1 to 4.8)	-0.3 (-6.8 to 6.3)	-1.2 (-5.2 to 2.9)
**Change in total cholesterol – mmol/l**	0.2 (-0.2 to 0.6)	0.6 (0.1 to 1.1)	0.1 (-0.3 to 0.4)	0.1 (-0.2 to 0.3)
**Change in LDL cholesterol – mmol/l**	0.2 (-0.1 to 0.5)	0.3 (-0.1 to 0.8)	0.0 (-0.3 to 0.2)	0.0 (-0.2 to 0.2)
**Change in HDL cholesterol – mmol/l**	0.0 (-0.1 to 0.1)	0.0 (-0.0 to 0.2)	0.1 (-0.0 to 0.2)	0.1 (-0.0 to 0.1)
**Change in triglycerides – mmol/l**	-0.2 (-0.7 to 0.4)	1.2 (0.9 to 1.6)	0.0 (-0.3 to 0.4)	0.1 (-0.1 to 0.4)

[a] Changes relative to baseline in primary and secondary endpoints in response to BSE or placebo, respectively, in participants with SIRD-like (n=9 placebo and 8 BSE), MOD-like (n=10 placebo and 3 BSE) and MARD-like (n=20 placebo and 24 BSE).

[b] Estimated mean differences of values in response to BSE minus placebo are presented as means with 95% confidence intervals.

[c] The body-mass index (BMI) is the weight in kilograms divided by the square of the height in meters.

[d] The fatty liver index was calculated based on BMI, waist circumference, triglycerides and GGT.

Supplementary Figures

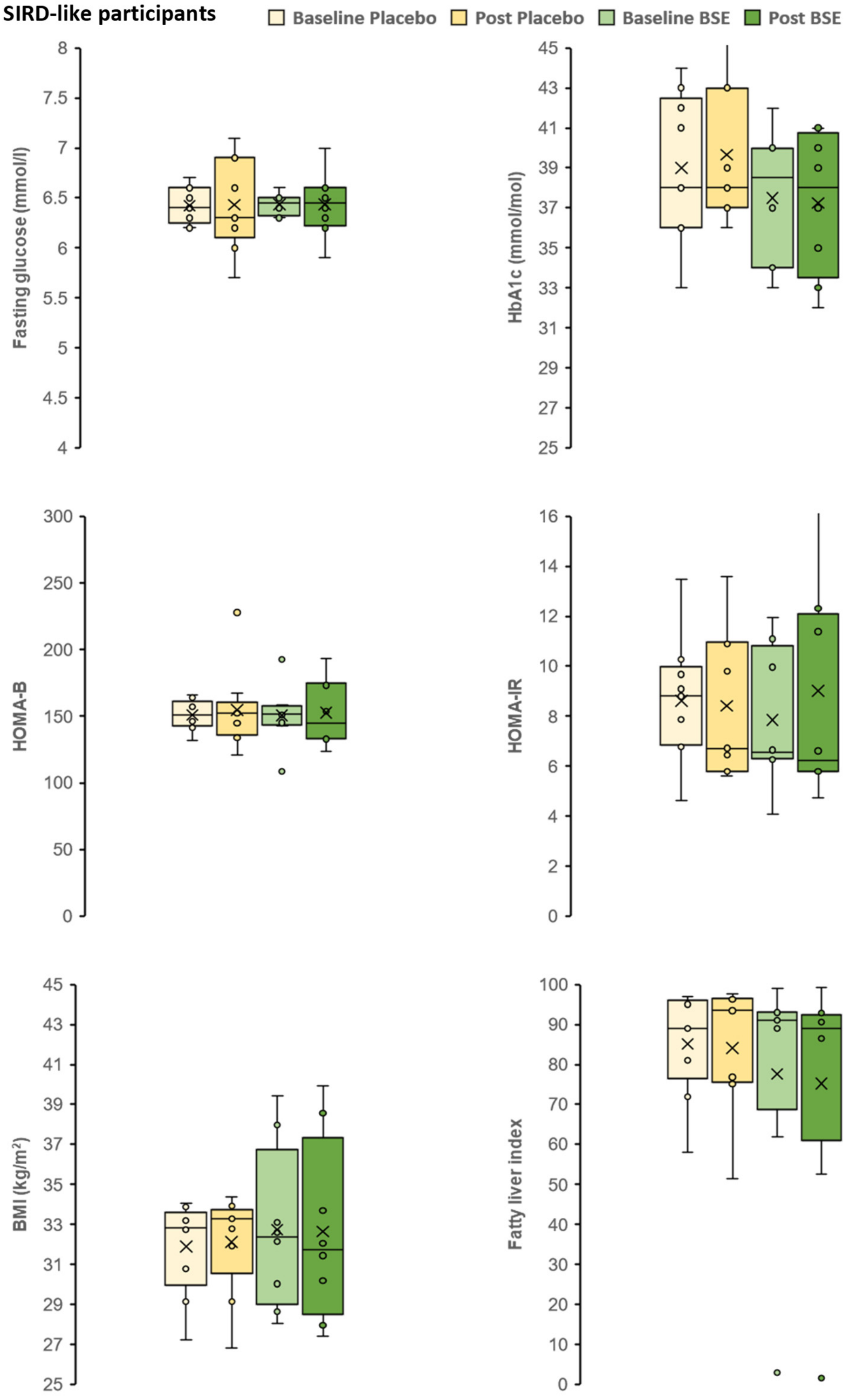

Supplementary Figure 1. Distribution of primary and secondary variables in SIRD-like participants.

Box plots show individual data points, medians (straight lines inside box) and means (cross marking) with hinges representing lower and upper quartile. Data are from baseline and post-treatment in the placebo (n=9) and BSE (n=8) groups of SIRD-like participants. Individuals with similar values are represented by the same circle.

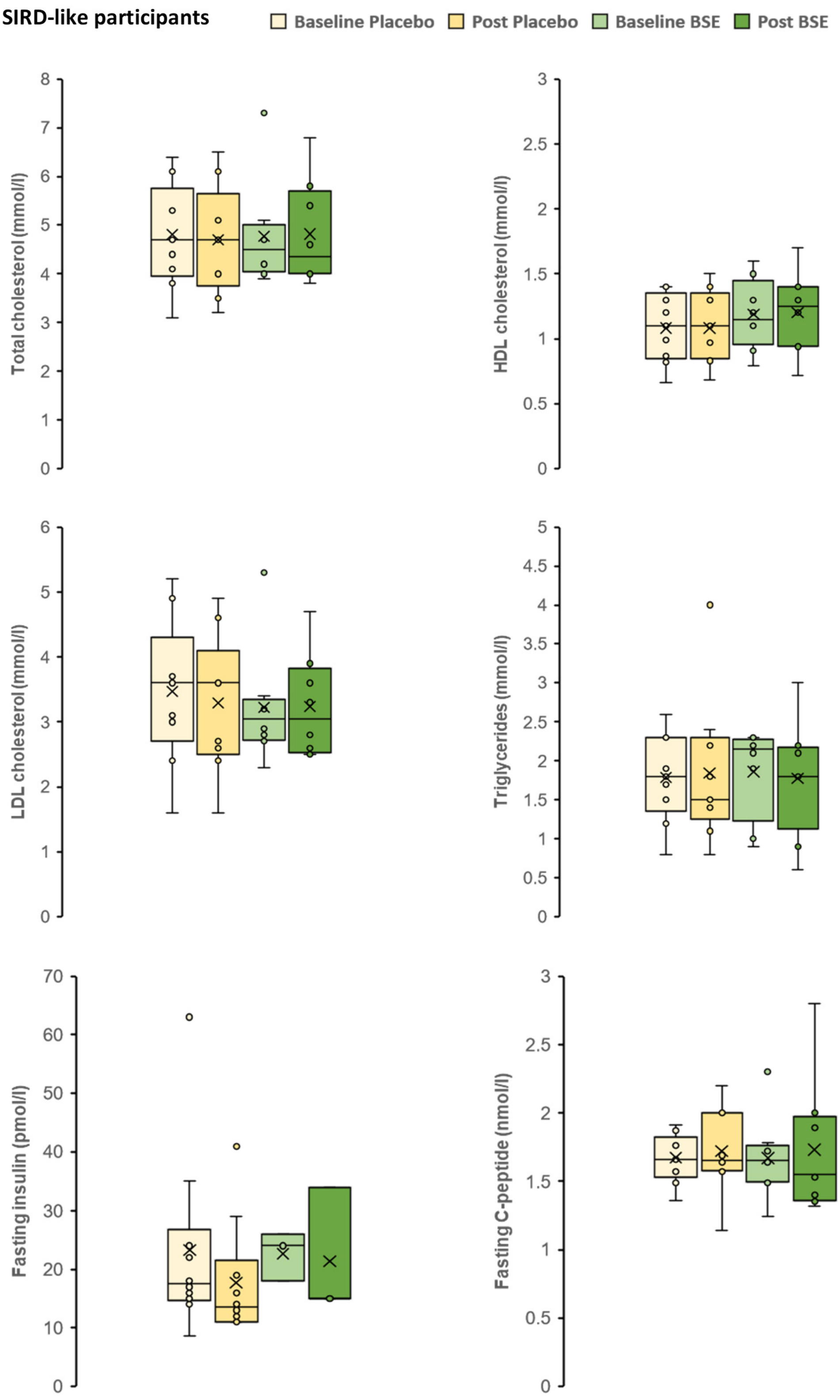

Supplementary Figure 2. Distribution of cholesterol, triglycerides, insulin and C-peptide in SIRD-like participants.

Box plots show individual data points, medians (straight lines inside box) and means (cross marking) with hinges representing lower and upper quartile. Data are from baseline and post-treatment in the placebo (n=9) and BSE (n=8) groups of SIRD-like participants. Individuals with similar values are represented by the same circle.

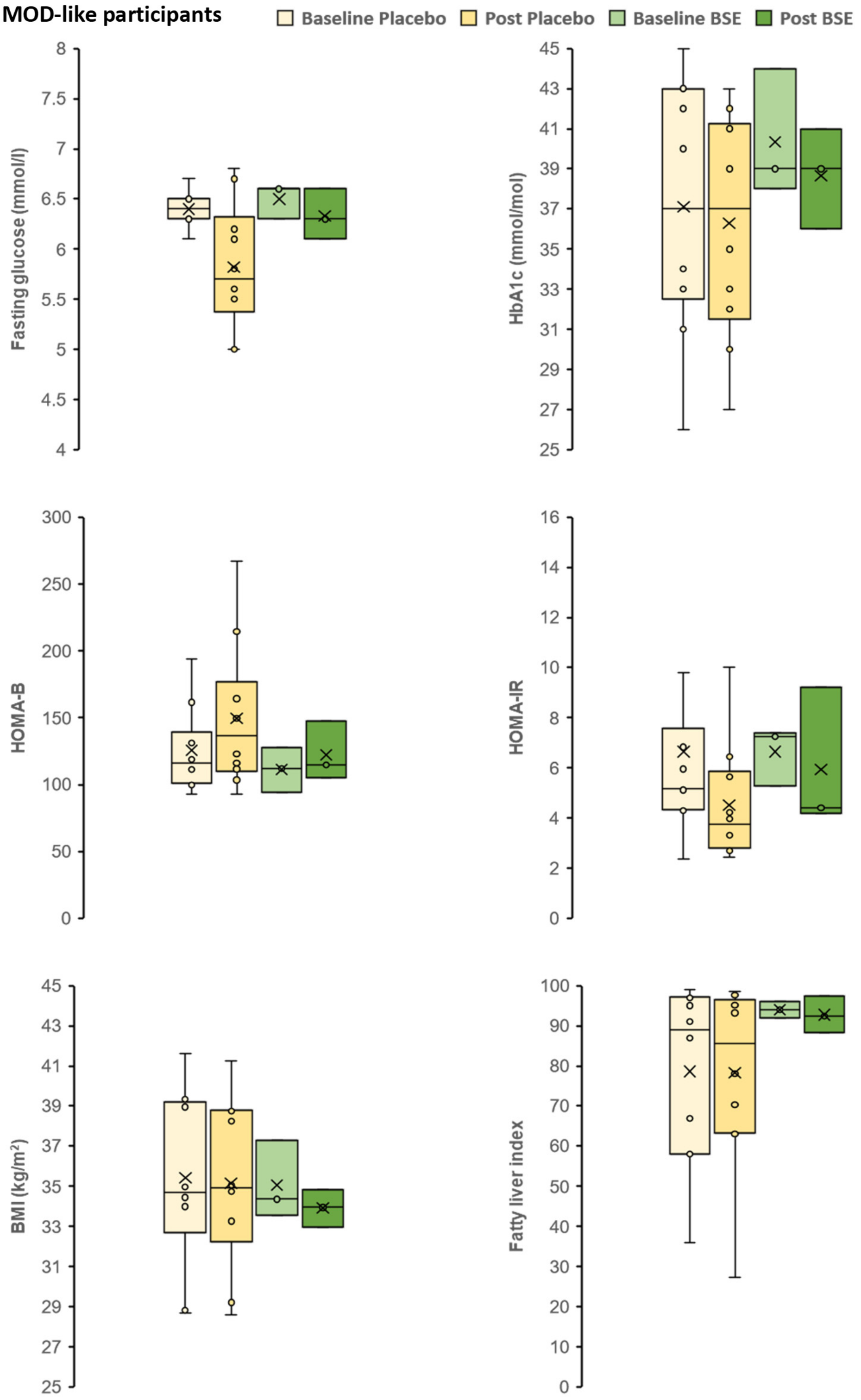

Supplementary Figure 3. Distribution of primary and secondary variables in MOD-like participants.

Box plots show individual data points, medians (straight lines inside box) and means (cross marking) with hinges representing lower and upper quartile. Data are from baseline and post-treatment in the placebo (n=10) and BSE (n=3) groups of MOD-like participants. Individuals with similar values are represented by the same circle.

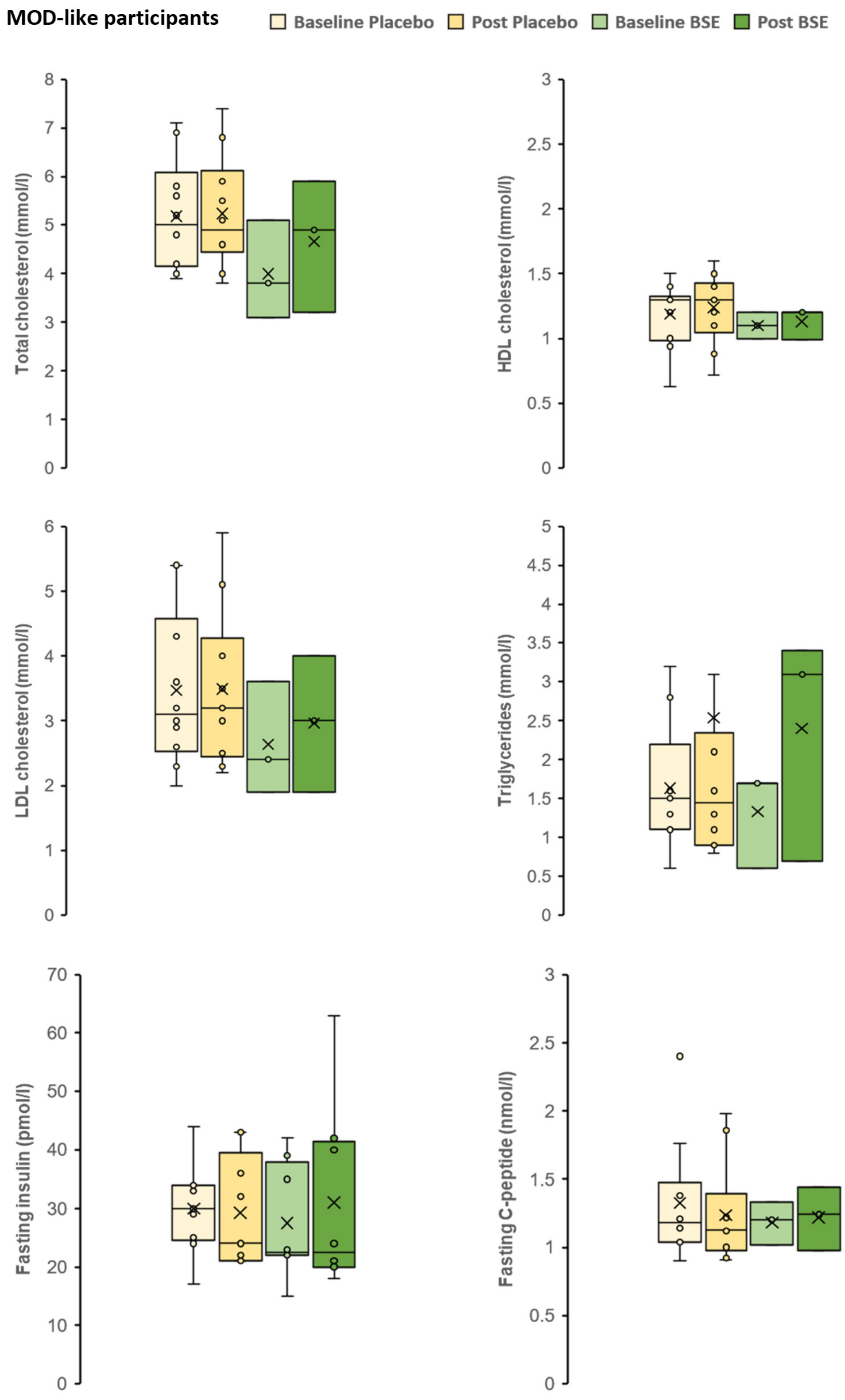

Supplementary Figure 4. Distribution of cholesterol, triglycerides, insulin and C-peptide in MOD-like participants.

Box plots show individual data points, medians (straight lines inside box) and means (cross marking) with hinges representing lower and upper quartile. Data are from baseline and post-treatment in the placebo (n=10) and BSE (n=3) groups of MOD-like participants. Individuals with similar values are represented by the same circle.

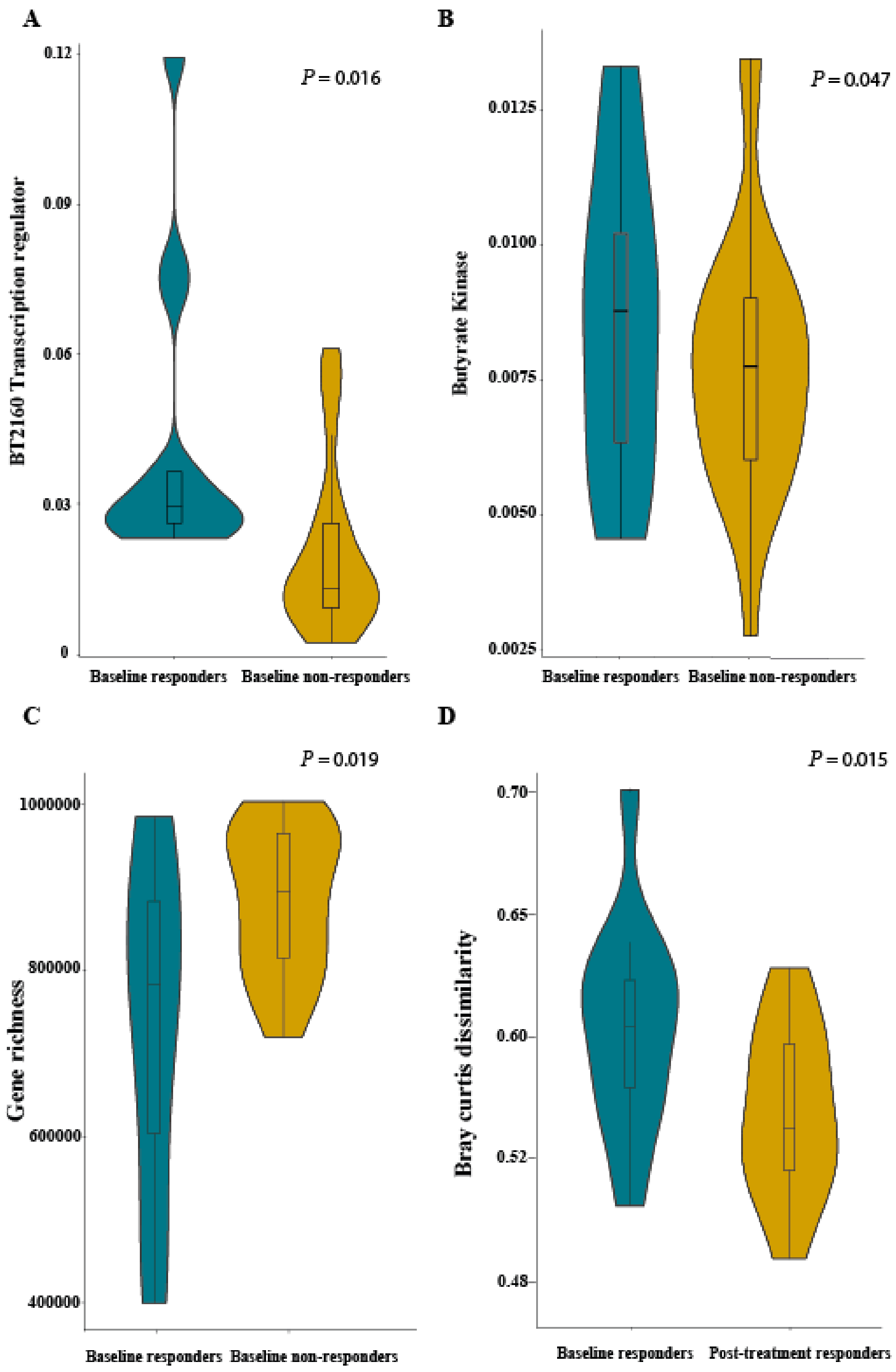

Supplementary Figure 5. Gut microbiota profiles of responders and non-responders to BSE.

a,b, Violin plots showing the relative abundance at baseline of (**a**) the transcriptional regulator of the operon, BT2160 (permutation test *P*=0.02; two-sided test) and (**b**) butyrate kinase (permutation test *P*=0.047; two-sided test) in participants with a pronounced response ("responders"; n=13) and less pronounced response to BSE (n=22), respectively. **c**, Gene richness at baseline between participants with a pronounced response (n=13) and less pronounced response (n=22) to BSE treatment (Wilcox rank-sum test *P*=0.02; two-sided test). **d**, Boxplots of Bray-Curtis dissimilarity at species level in the gut microbiota of the pronounced responders at baseline and post-treatment (Wilcox rank-sum test

P=0.02; two-sided test; n=13). The boxes mark the lower and upper quartiles with the median indicated by a line. The whiskers denote the lower adjacent value (the smallest value larger than or equal to the first quartile minus 1.5 times the interquartile range) and the upper adjacent values (the largest value less than or equal to the upper quartile plus 1.5 times the interquartile range), respectively. The ends of the violin plots denote the minimum and maximum values. The width of the violin plots denotes the probability distribution for the values.

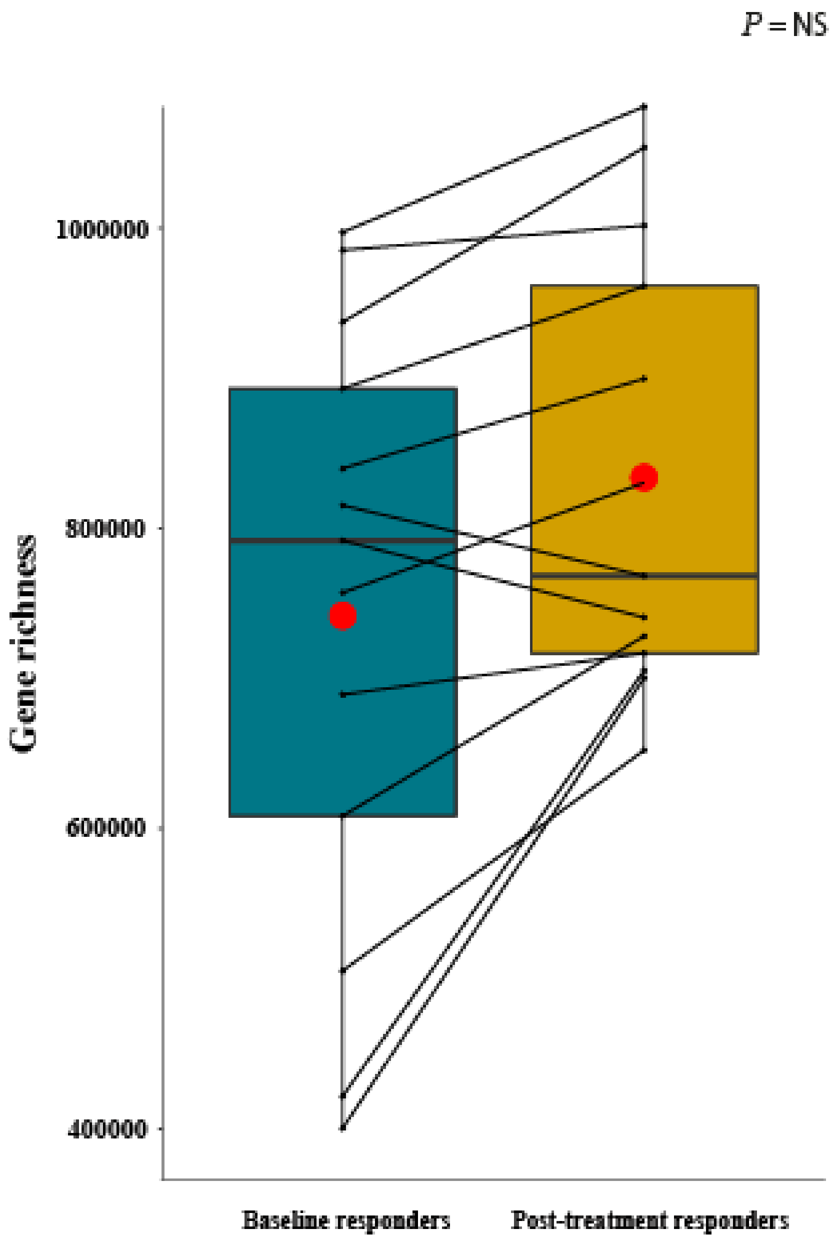

Supplementary Figure 6. Gene richness in the gut microbiota at baseline and post-treatment in pronounced responders.

Gene richness at baseline and post-treatment in the participants with a pronounced response to BSE (n=13 participants with a pronounced response with paired comparisons between values at baseline and after treatment). The lines reflect the change of gene richness of each participant. The median is indicated by the straight lines in the box plots, the red indicator is the mean, while the lower and upper hinges represent the first and third quartiles. The whiskers extend from the hinges to the largest and smallest value 1.5 times the inter-quartile range.

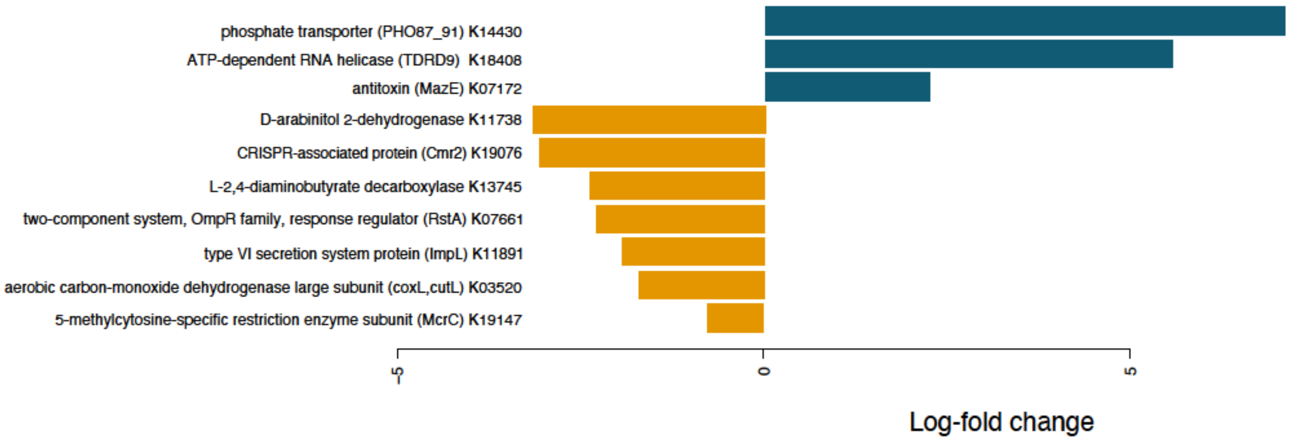

Supplementary Figure 7. Altered genes between participants with pronounced and less pronounced response to BSE.

In addition to the changed abundance of BT2160 (Supplementary Figure 6a), a number of other genes were different at baseline (at adjusted P<0.05) between participants with a pronounced (green) and less pronounced response (yellow) to BSE (with a pronounced response defined as a reduction of fasting blood glucose greater than the top quartile of glycemic improvement [0.3 mmol/l]).

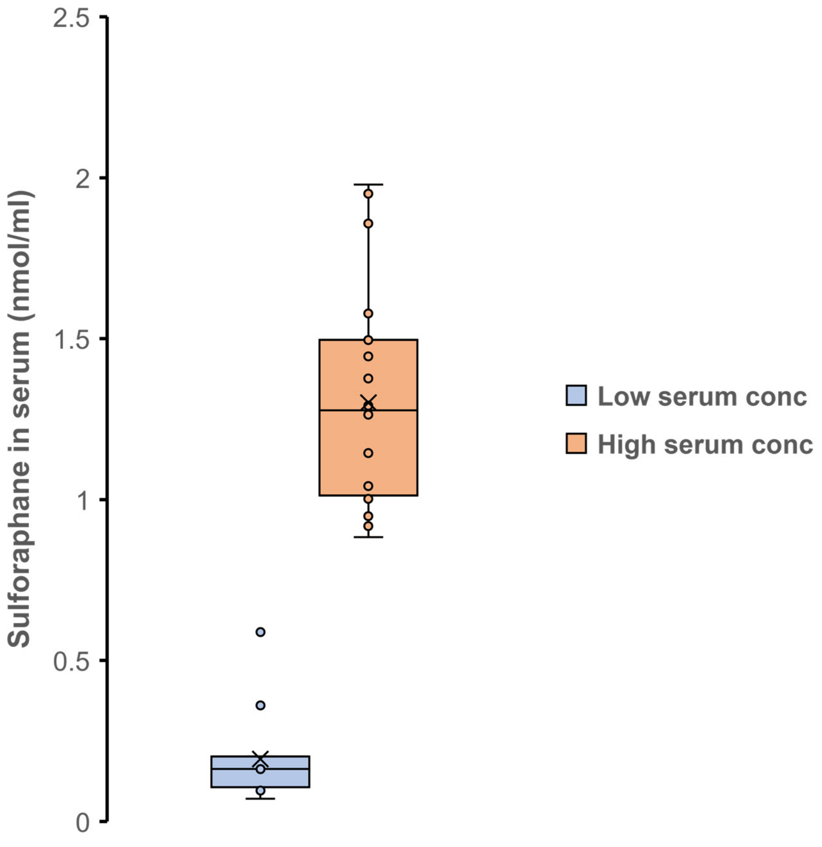

Supplementary Figure 8. Sulforaphane concentration in serum in BSE-treated participants.

The serum sulforaphane concentration had a bimodal distribution in the participants with a mean concentration of 0.2 nmol/ml at the low end (shown in the blue box plot; n=11 serum samples from different participants) and 1.3 nmol/ml at the high end (shown in the orange box plot; n=24 serum samples from different participants). The mean values differed by approximately twice the common standard deviation. The box plots show individual data points, medians (straight lines inside box) and means (cross marking) with hinges representing lower and upper quartile of sulforaphane concentration. Individuals with similar values are represented by the same circle.

Supplementary References

1. Ahlqvist, E., *et al. Lancet Diabetes Endocrinol* **6**, 361-369 (2018).

2. Zaharia, O.P., *et al. Lancet Diabetes Endocrinol* **7**, 684-694 (2019).

3. Axelsson, A.S., *et al. Sci Transl Med* **9**(2017).
