# Supplementary material for: Effect of broccoli sprout extract and baseline gut microbiota on fasting blood glucose in prediabetes: a randomized, placebo-controlled trial
Source: Nat Microbiol. 2025 Feb 10;10(3):681–93. doi: 10.1038/s41564-025-01932-w (PMC11879859; doi:10.1038/s41564-025-01932-w)
Supplement: Supplementary file 2 — Reporting Summary [file 41564_2025_1932_MOESM2_ESM.pdf]

nature portfolio

Corresponding author(s): Rosengren Anders

Last updated by author(s): Dec 20, 2024

Reporting Summary

Nature Portfolio wishes to improve the reproducibility of the work that we publish. This form provides structure for consistency and transparency in reporting. For further information on Nature Portfolio policies, see our Editorial Policies and the Editorial Policy Checklist.

Statistics

For all statistical analyses, confirm that the following items are present in the figure legend, table legend, main text, or Methods section.

n/a	Confirmed	
☐	☒	The exact sample size (*n*) for each experimental group/condition, given as a discrete number and unit of measurement
☒	☐	A statement on whether measurements were taken from distinct samples or whether the same sample was measured repeatedly
☐	☒	The statistical test(s) used AND whether they are one- or two-sided<br>*Only common tests should be described solely by name; describe more complex techniques in the Methods section.*
☐	☒	A description of all covariates tested
☐	☒	A description of any assumptions or corrections, such as tests of normality and adjustment for multiple comparisons
☐	☒	A full description of the statistical parameters including central tendency (e.g. means) or other basic estimates (e.g. regression coefficient) AND variation (e.g. standard deviation) or associated estimates of uncertainty (e.g. confidence intervals)
☐	☒	For null hypothesis testing, the test statistic (e.g. *F*, *t*, *r*) with confidence intervals, effect sizes, degrees of freedom and *P* value noted<br>*Give P values as exact values whenever suitable.*
☒	☐	For Bayesian analysis, information on the choice of priors and Markov chain Monte Carlo settings
☒	☐	For hierarchical and complex designs, identification of the appropriate level for tests and full reporting of outcomes
☒	☐	Estimates of effect sizes (e.g. Cohen's *d*, Pearson's *r*), indicating how they were calculated

Our web collection on statistics for biologists contains articles on many of the points above.

Software and code

Policy information about availability of computer code

Data collection

Provide a description of all commercial, open source and custom code used to collect the data in this study, specifying the version used OR state that no software was used.

Data analysis

Statistical analyses were performed using SPSS (version 26, IBM) or R 4.1.0. The package XGBoost version 1.6.0.1 was used in R 4.1.0. Principal coordinates analysis was performed on Bray-Curtis dissimilarity on species level, calculated based on species abundances, and significance was determined by PERMANOVA test using the adonis2 function with 10,000 permutations. Significantly differential abundant species tables were obtained using the deseq2 package with adjustment for subjects at different visits. Correlation of gut microbiota species abundances with clinical parameters was performed using distance-based redundancy analysis with the capscale function using anova.cca and 10,000 permutations. The functions utilized in these analyses are implemented in the vegan package (Community Ecology Package-R package version 1.17-8). All statistical analyses involving fecal whole genome metagenomics were performed in R 4.1.0. All R packages used were such that are publically available.

For manuscripts utilizing custom algorithms or software that are central to the research but not yet described in published literature, software must be made available to editors and reviewers. We strongly encourage code deposition in a community repository (e.g. GitHub). See the Nature Portfolio guidelines for submitting code & software for further information.

Data

Policy information about availability of data

All manuscripts must include a data availability statement. This statement should provide the following information, where applicable:

- Accession codes, unique identifiers, or web links for publicly available datasets
- A description of any restrictions on data availability
- For clinical datasets or third party data, please ensure that the statement adheres to our policy

Raw metagenomic sequence data have been deposited in the EMBL-EBI European Nucleotide Archive (ENA) under accession number PRJEB77105. To remove human contamination, reads were mapped against human genome (hg19) using Bowtie2 v2.4.4. Filtered reads passing the quality criteria were then mapped using Kraken2 with default settings against the RefSeq database (release 107). The BT2156-BT2160 protein sequences were downloaded from refseq [WP_008763945-WP_00876394] and mapped against Bacteroides D2 [accession id NZ_CP102261] and Bacteroides DM10 [accession id CP060488] based on the reference genomes of the species used in the RefSeq database (release 107). All clinical data supporting the findings of this study are available as Supplementary Data and as source data online via the publisher's website. Further data requests should be submitted to the corresponding author. De-identified individual and/or study-level data will be shared with researchers who provide a methodologically sound proposal and if regulatory criteria are met. Data will be available immediately after publication of this study (no end date). Access to anonymized data may be granted following review (time frame <20 office days) to ensure compliance with relevant ethical and legal considerations. The study protocol is appended with the paper and available online. Source data are provided with the paper.

Research involving human participants, their data, or biological material

Policy information about studies with human participants or human data. See also policy information about sex, gender (identity/presentation), and sexual orientation and race, ethnicity and racism.

Reporting on sex and gender	The study enrolled both women and men, and the results are applicable to both women and men. Gender distribution is presented for data on enrollment and treatment allocation.Gender was determined by self-report and the social security number (no discrepancies).
Reporting on race, ethnicity, or other socially relevant groupings	The study includes participants living in western Sweden in both rural and urban areas and a wide distribution of socioeconomic background.
Population characteristics	Participants had fasting blood glucose at 6.1-6.9 mmol/l, no previous diabetes, age 35-75 years and BMI 27-45 kg/m2.
Recruitment	A random selection of members of the general population aged 35-75 years in Gothenburg, Sweden, and surrounding municipalities received letters with study information and they responded to invitation by self-selection. The self-selection procedure may result in a selection of individuals that are not entirely representative of the full population of pre-diabetic individuals. The randomized treatment allocation should however minimize any systematic differences between randomization groups. All participants provided written informed consent before inclusion.
Ethics oversight	The protocol was approved by the Regional Ethics committee of Gothenburg (433-18).

Note that full information on the approval of the study protocol must also be provided in the manuscript.

Field-specific reporting

Please select the one below that is the best fit for your research. If you are not sure, read the appropriate sections before making your selection.

☒ Life sciences ☐ Behavioural & social sciences ☐ Ecological, evolutionary & environmental sciences

For a reference copy of the document with all sections, see nature.com/documents/nr-reporting-summary-flat.pdf

Life sciences study design

All studies must disclose on these points even when the disclosure is negative.

Sample size	The study was designed to have 80% power to detect a treatment effect of 0.3 mmol/l between BSE and placebo. The standard deviation of change in fasting blood glucose over 12 weeks is 0.63 mmol/l, based on analyses in our longitudinal cohorts of subjects with impaired fasting blood glucose. At alpha 0.05, at least 74 study participants were needed.
Data exclusions	The full analysis set includes all participants who have a measurement of fasting glucose after randomization, independent of compliance. Fifteen participants were lost to follow-up (9 assigned to BSE and 6 to placebo).
Replication	The distribution of clusters was replicated in a separate cohort. The findings of metagenomic and clinical data were analysed in one study cohort but with several approaches to corroborate the general conclusions. The findings in the present study cohort will have to be replicated in future trials.
Randomization	The randomization (in a 1:1 ratio between BSE and placebo) was generated by independent statisticians using a computer-based block randomization algorithm with balanced blocks. Allocation was concealed (via sealed envelopes) to both participants and study personnel until

	end of the study. Thus, the generation of the random sequence, participant enrolment by study personnel, and the allocation to randomization groups were clearly separated.
Blinding	The study was double-blind. The placebo looked, smelled and tasted similar to the active compound and had the same constituents except BSE.

Reporting for specific materials, systems and methods

We require information from authors about some types of materials, experimental systems and methods used in many studies. Here, indicate whether each material, system or method listed is relevant to your study. If you are not sure if a list item applies to your research, read the appropriate section before selecting a response.

Materials & experimental systems

n/a	Involved in the study
☒	☐ Antibodies
☒	☐ Eukaryotic cell lines
☒	☐ Palaeontology and archaeology
☒	☐ Animals and other organisms
☐	☒ Clinical data
☒	☐ Dual use research of concern
☒	☐ Plants

Methods

n/a	Involved in the study
☒	☐ ChIP-seq
☒	☐ Flow cytometry
☒	☐ MRI-based neuroimaging

Clinical data

Policy information about clinical studies

All manuscripts should comply with the ICMJE guidelines for publication of clinical research and a completed CONSORT checklist must be included with all submissions.

Clinical trial registration	NCT03763240
Study protocol	Appended with the paper
Data collection	The study was conducted at Gothia Forum, Sahlgrenska University hospital, Gothenburg, Sweden. Study recruitment and data collection were done in 2018-2020.
Outcomes	The primary variable was the change in fasting blood glucose at final visit (visit 3) relative to baseline (visit 2). The secondary variables were the change from baseline in HbA1c, BMI, insulin resistance (measured by HOMA-IR), insulin secretion (measured by HOMA-B), fasting blood lipids, and a fatty liver index based on BMI, waist circumference, triglycerides and GGT. Liver parameters, including GGT, ALP, AST, ALT and bilirubin were also measured, and hemoglobin, thrombocytes, thyroid-stimulating hormone, creatinine and estimated glomerular filtration rate (based on creatinine) were analyzed as safety variables.

Plants

Seed stocks	Broccoli sprout extracts were prepared from raw broccoli sprouts by Lantmännen AB, Sweden.
Novel plant genotypes	N/A
Authentication	N/A
