# Supplementary material for: Effect of broccoli sprout extract and baseline gut microbiota on fasting blood glucose in prediabetes: a randomized, placebo-controlled trial
Source: Nat Microbiol. 2025 Feb 10;10(3):681–93. doi: 10.1038/s41564-025-01932-w (PMC11879859; doi:10.1038/s41564-025-01932-w)
Supplement: Supplementary file 3 — Study protocol and statistical analysis plan. [file 41564_2025_1932_MOESM3_ESM.pdf]

Effect of BSE on blood glucose

Protocol number: **3**

Date: **26 Februari 2019**

STUDY ADMINISTRATION

Table 1. Study administration

**Function**	**Responsible person**
Sponsor and Principal Investigator	Anders Rosengren, MD PhD<br>Institutionen för neurovetenskap och fysiologi, Box 432, 40530 Göteborg<br>Tel: 070-5316704<br>Email: anders.rosengren@gu.se
Study location	Gothia Forum, Gröna stråket 12 (Sahlgrenska University Hospital), 413 45 Göteborg, Sweden

PRINCIPAL INVESTIGATOR SIGNATURE

This study will be conducted in accordance with the study protocol and relevant laws.

Anders Rosengren	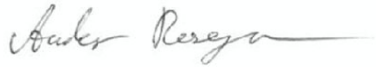	2019-02-26
Sponsor and Principal Investigator	Signature	Date

SYNOPSIS

**Study name**	Effect of BSE on blood glucose
**Protocol number**	**3**
**Study location**	Gothia Forum, Gröna stråket 12 (Sahlgrenska University Hospital), 413 45 Göteborg, Sweden
**Number of subjects**	100
**Study design**	Randomized double-blinded parallel arm
**Study population**	Individuals with pre-diabetes
**Aims**	The aim of the study is to measure the effect of broccoli sprout extract (BSE) on fasting glucose
**Summary of study plan**	Here we will investigate the effect of sulforaphane, provided as a broccoli sprout extract (BSE) on blood glucose in pre-diabetic individuals without metformin treatment. This will address whether BSE could be used to improve glucose control in drug-naïve pre-diabetic individuals. The participants will receive BSE or placebo in a randomized double-blind parallel arm study. The participants will take their study compound once daily over 12 weeks. The primary study variable is fasting glucose.
**Duration of study**	Aug 2018- Dec 2020

1. BACKGROUND

Type 2 diabetes (T2D) is an escalating health problem of enormous proportions. An alarming 350 million persons have T2D, and the number is expected to exceed 500 million by 2030. The disease poses a heavy burden on health care systems in both developed and developing countries. The disease leads to devastating complications in kidneys, eyes and the cardiovascular system.

T2D results from a combination of insufficient insulin secretion from pancreatic islets and insulin resistance of target cells. T2D develops slowly and there is a large number of people in the general population that are at risk for T2D. The people from the general population that have progressed farthest towards T2D are said to have 'pre-diabetes', defined as impaired fasting glucose or impaired glucose tolerance. Lifestyle changes, including dietary interventions, is a cornerstone to counteract the progression of prediabetes to overt T2D. However, many people find it difficult to know what to eat, because of conflicting reports, and there are many well-studied psychological reactions that make lifestyle changes difficult, including lack of motivation, loss aversion (aversion to give up established lifestyles), and automatic behaviour.

Metformin is first-in-line treatment for newly diagnosed T2D. However, 15% of all T2D patients cannot take metformin because of reduced kidney glomerular filtration rate and hence increased risk of lactic acidosis. Moreover, many patients treated with metformin develop nausea, bloating, abdominal pain, or diarrhea, and another 5 to 10% of the patients are unable to continue with metformin because of the gastrointestinal problems. This also limits the use of metformin as a long-term treatment for preventing T2D.

Clearly, new options are needed to more effectively prevent T2D. Since diet is so central, new food supplements ("functional food") could be one potential avenue, as they may have fewer side effects and be more easily tolerated. A limitation to current functional foods is lack of understanding of the mechanism of action and insufficient scientific effect evaluation.

We have recently identified sulforaphane as a new anti-diabetic compound that counteracts exaggerated hepatic glucose production in obese T2D patients (Axelsson et al., *Science Translational Medicine*, 2017). The identification was made possible by analysing genetic and gene expression data from liver tissue to construct a 'disease signature' of 50 genes that are perturbed in patients with T2D. We then compiled a library of 3800 'drug signatures', using publically available expression data (www.ncbi.nlm.nih.gov/geo). A drug signature represents the genes for which the expression is significantly affected by a specific compound. The drug signatures were matched with the disease signature to find compounds that could potentially reverse the aberrantly expressed disease genes (overexpressed T2D genes should be downregulated in the drug signature and vice versa).

From these analyses, sulforaphane stood out as the highest ranked compound. Sulforaphane is highly contained in broccoli sprout extract, BSE, and has previously been studied clinically for cancer prevention but has not been implicated in T2D. We first observed that the

compound suppressed glucose production from hepatic cells by nuclear translocation of the transcription factor NRF2. This led to decreased expression of key enzymes in gluconeogenesis. Next, the compound was shown to reduce gluconeogenesis and improve glucose intolerance in diabetic animals by a magnitude similar to that of metformin. The treatment also reversed the 50-gene disease signature in the liver from these animals.

Finally, we translated the findings to patients with T2D. In collaboration with Lantmännen (the Swedish farmer's association) we generated a water-soluble powder of broccoli sprout extracts (BSE) containing high concentrations of sulforaphane. In mice, BSE, provided as gavage, improved glucose tolerance to the same extent as HPLC-purified sulforaphane. Inactivation of sulforaphane in the BSE by boiling abolished the effect, demonstrating that sulforaphane is the active component in this case.

We then recruited 97 patients with T2D and metformin treatment who received BSE (with 150 µmole sulforaphane) or placebo (randomized double-blind) once daily over 12 weeks. In obese patients (BMI>30 kg/m^2) with dysregulated T2D, BSE improved HbA1c by 4 mmol/mol ($p<0.05$) and fasting glucose by 0.9 mM ($p<0.05$). The effect size is in the same range as that seen by recently approved anti-diabetic DPPIV-inhibitors. BSE had no severe adverse effects in this study, in agreement with previous studies.

2. RATIONALE FOR THE STUDY AND STUDY DESIGN

Here we will extend on our previous study by investigating the preventative effects of BSE with standardized amounts of sulforaphane (at least 150 µmole daily) in pre-diabetic individuals. This will address whether BSE could be used to improve glucose control in drug-naïve pre-diabetic individuals.

BSE would be an attractive food supplement to reduce blood glucose for several reasons. 1) We have extensive data from diabetic animal models and T2D patients that BSE improves fasting glucose. 2) Hepatic glucose production is typically exaggerated in individuals at risk for T2D and it is a central pathophysiological process that leads to increased fasting glucose and progression to T2D. We have characterized the mechanism of action of sulforaphane in the BSE and shown that it affects the anti-oxidative NRF2 system, and leads to reduced expression of gluconeogenetic enzymes and hence suppressed hepatic glucose production. The compound therefore counteracts a key mechanism leading to T2D. 3) Metformin, which also reduced hepatic glucose production (although via different mechanisms), has side effects that means that all people cannot tolerate metformin. BSE have few adverse effects. BSE is being tested for the treatment or prevention of cancer and inflammatory diseases in ~30 clinical trials without any serious adverse events reported. Moreover, cruciferous vegetables, including broccoli sprouts, are regular dietary components in many regions of the world. Based on the widespread use of broccoli sprouts as a food and previous human studies with BSE, including our own recent study, no severe or serious risks are anticipated to be associated with the administration of BSE. The low toxicity makes BSE ideal as a food supplement for reducing blood glucose and prevent T2D.

We aim to study the effect of BSE with standardized amounts of sulforaphane on fasting glucose in pre-diabetic individuals, here defined as having impaired fasting glucose. Based on the mechanism of action of sulforaphane, we focus on individuals with impaired fasting glucose rather than impaired glucose tolerance (i.e. elevated glucose levels after glucose ingestion). Our previous characterization of the mechanism of action also enables us to focus on the most relevant variable, in this case fasting glucose, which consequently will be the primary study variable.

BSE is a freeze-dried powder of an aqueous extract of broccoli sprouts that provides a consistent and stable source of sulforaphane. We will use a parallel arm study design with 50 subjects receiving BSE and 50 receiving placebo. The randomization will be double-blind. The study will be done at one centre. Subjects will be treated for 12 weeks.

3. PROJECT DESCRIPTION

3.1 Recruitment

A random selection of members of the general population aged 35-75 years in Göteborg and surrounding municipalities, who have registered addresses and Swedish personal numbers, will receive an invitation letter with study information. The letter will be sent by regular post via PostNord. The addresses will be retrieved from the general population address registry. This is a large and socially heterogeneous area, which means that the invitation letters will reach a broad spectrum of the general population.

Interested participants go to a dedicated study website, which has a simple interface for booking a time for a screening visit. The website has the necessary IT security and the participants will not be able to see which other people have booked a time (they will only see available and non-available slots).

They enter their name, telephone number and email address. They receive a confirmation email with instructions for the screening visit.

It is not a prerequisite to have received the letter to participate, which means that people who get to know about the study through other channels (e.g. media coverage, word of mouth) will also be able to participate in the study.

It is possible to re-screen a person who at some occasion has had pre-diabetic glucose values but because of variation in fasting glucose was not included in the first assessment (screening failure at screening visit or visit 2). Anyone who is re-screened will start over again with a new written informed consent, a new screening number and the person has to pass the study criteria at both the new screening visit and visit 2 to be randomized.

3.2 Study criteria

Inclusion criteria

- Impaired fasting glucose, defined as fasting blood glucose 6.1-6.9 mM.
- Written informed consent
- Age 35-75 years. Participating women of fertile age must have no current pregnancy, which will be assessed by pregnancy test.
- Body mass index 27-45 kg/m^2

Exclusion criteria

- Diagnosed with diabetes mellitus according to the WHO criteria
- Anti-diabetic medication
- Active liver disease

- At screening or at any subsequent visit a level of aspartate aminotransferase (ASAT) or alanine aminotransferase (ALAT) of more than three times the upper limit of the normal range
- Gastrointestinal ailments which may interfere with the ability to adequately absorb sulforaphane
- At screening visit creatinine > 130 µmol/L
- Coagulation disorder or current anti-coagulant therapy, which may be affected by the BSE
- Diagnosed with a cardiovascular disease or known cardiovascular event, transient ischemic attack, coronary by-pass surgery or other coronary vessel intervention within 6 months prior to enrolment
- Systemic glucocorticoid treatment
- Herbal treatment, defined as food supplement (except multivitamin treatment) with herbal or vegetable extracts that may affect blood glucose
- Allergy to broccoli
- Participant unable to understand the study information
- Participation in other clinical trial which may affect the outcome of the present study
- Any other physical or psychiatric condition or treatment that in the judgment of the investigator makes it difficult to participate in the study.

Criteria for withdrawal

- The participant can withdraw from the study at any time without further motivation and without any consequences for his/her future treatment.
- The participant will be withdrawn should they develop any condition or begin taking any compound that is part of the exclusion criteria.
- A participant can be withdrawn if he/she does not adhere to the procedures as specified in the protocol. No extra financial or treatment compensation is given to the participant in that case.
- If the participant experience serious adverse events, and there are reasons to think that the event is related to the study, he/she will be immediately withdrawn and prompt action will be taken to assist the participant as necessary.
- The study can be stopped at any time due to a lack of treatment effect, side effects or that the procedures of the protocol are not adhered to. In that case all participants as well as relevant authorities will be notified.

All participants that are withdrawn will continue their regular medication and their ordinary physician will be notified as appropriate.

3.3 Screening visit

Participants who have booked a time for a screening visit are instructed (by a confirmation email) not to conduct intense physical activity or drink alcohol 24 h before the visit. They should be fasting since midnight. Nicotine users should not have used nicotine the same day.

At the screening visit the study personnel first informs the participants (in group) on what it means to participate in the study (this has also been described in the initial participant information sent by post). The participants who are interested in taking part then meet a member of the personnel on their own where they can ask questions and study criteria are being checked. Participants who sign a written informed consent will then undergo capillary fasting blood glucose measurement.

The capillary fasting blood glucose is analysed by a glucometer on-site. If the capillary fasting blood glucose is 6.0-6.9 mmol/l we proceed with venous blood sampling to analyse the study criteria, as well as weight and length measurements. If it is obvious that the participant will not fulfil the study criteria (based on capillary blood glucose measurement or the study criteria that we can assess without blood analyses) we do not proceed with venous blood sampling.

The participant gets a screening number (S1, S2, etc.). The code list for screening number and screening ID is kept with the Investigator. Those who come to a screening visit but are not eligible to participate will continue to be managed by their regular physician. If blood glucose is 7.0 mM or above (which means risk for diabetes diagnosis, which has to be confirmed by a second glucose analysis), the individual will be referred to primary healthcare to assess whether they have overt diabetes.

The following will be done at the screening visit:

- The participant will receive information on the study in group
- Each participant meets a member of the study staff on their own and can ask questions
- Written informed consent is signed if the participant wants to take part
- ID is checked
- Information on current use of pharmaceutical drugs is collected
- Inclusion and exclusion criteria are checked
- The participant gets a screening number.
- Capillary fasting blood glucose is analysed on-site.

If capillary fasting blood glucose is 6.0-7.9 mM we proceed with the following:

- Length, weight, and waist-hip circumference are measured.
- Venous fasting blood samples are drawn for analysis of glucose, creatinine, ASAT, ALAT, GT, ALP, bilirubin, PK, thrombocytes. Total blood volume is estimated to 25 mL.
- If venous fasting blood glucose is above or equal to 6.1 mmol/l and below 7.0 mmol/l, the participant is scheduled for visit 2, which will be 1-14 days after

the screening visit.

- They receive a tube for stool sampling and are instructed how to sample.

3.4 Visit 2

Participants who are eligible to participate will be scheduled for a second visit 1-14 days after their first visit. If the venous analyses from the screening visit of creatinine, ASAT, ALAT, GT, ALP, bilirubin, PK, thrombocytes, show clinically relevant abnormalities that upon decision by PI precludes participation, that the subject's visit 2 will be cancelled.

The participants are recommended not to conduct intense physical activity or drink alcohol 24 h before the visit. They should be fasting since midnight. Nicotine users should not have used nicotine the same day.

The following will be done at visit 2:

- ID is checked
- Information on current use of pharmaceutical drugs is collected
- Venous fasting blood samples are drawn for analysis of fasting glucose, HbA1c, insulin, c-peptide, Hb, GT, lipid levels (HDL, LDL, total cholesterol, triglycerides), TSH and free fatty acids. Total blood volume is estimated to 25 mL.

If venous fasting blood glucose is 6.1-6.9 mM the individual can be included in the study and we proceed with the following:

- For women of fertile age we make a pregnancy test and proceed with the remaining points only if that is negative.
- The participant receives a study ID, using consecutive numbers and are randomized to receive BSE or placebo (see point 5.3 Randomization)
- Length, weight, and waist-hip circumference are measured.
- Participants complete a questionnaire on dietary habits, physical activity, smoking and alcohol habits.
- Participants leave their stool sample
- A note is made in the medical record about the visit.
- Study subjects receive their study medication (BSE or placebo) to bring home
- The receive a tube for stool sample to be sampled before visit 3
- Scheduled for visit 3. Visit 3 should be scheduled on the same weekday as visit 2, 12 (ideally) or 13 weeks later. If that is not possible, e.g. because of public holidays or unavailability of the participant to come on an assigned day, visit 3 will be scheduled as soon as possible 12 weeks after visit 2. Latest possible date for visit 3 is ~~14~~ 15 weeks after visit 2. If that is not possible to manage for the participant, he or she have to be excluded. The participant is instructed to contact us as soon as possible if he or she needs to change date for visit 3.

3.5 Inclusion

If the participant fulfil all study criteria they will be formally included. Included participant receive a study ID (1, 2, 3, etc.). The study ID will be different from the screening number (S1, S2, S3, etc.). The code list for study ID and personal ID is kept with the Investigator. Included participants will receive a card with information about the study and contact details to the study team. The card holds instructions to caregivers to contact the Investigator should the participant's medication be changed during the course of the study. The participant continues taking all their regular treatment during the study. Only coded data, based on the study number, will be used for analyses.

3.6 Between visit 2 and 3

Participants are instructed to take their BSE/placebo daily in the morning starting 12 weeks before visit 3. They have a paper attached on the box with their medication/placebo where they tick each day they have taken a dose. They will receive a slight surplus, in case they for some reason have to postpone visit 3. The reason is that we want to avoid gaps between the last intake and visit 3. If a participant or the study team with short notice have to cancel visit 3, e.g. due to illness, and the participant runs out of study medication we must have a buffer so that the treatment can continue daily until visit 3. The total treatment time can under no circumstances be more than 15 weeks, and if visit 3 has to be postponed beyond that limit the participant will be excluded. Any remaining doses should be brought back to the study team at visit 3.

The study team will contact the participants by phone 2-4 weeks after the initiation of the treatment to ask whether they experience any side effect and to check compliance. Moreover, information on changes in use of pharmaceutical drugs is collected. Reasonable efforts should be undertaken to get into contact with the participants by calling back if no reply, leaving messages and as a second option sending emails. Participants are also instructed to contact us in case they have any suspected side effects of the treatment.

3.7 Visit 3

The participants are recommended not to conduct intense physical activity or drink alcohol 24 h before the visit. They should be fasting since midnight. Nicotine users should not have used nicotine the same day.

The following procedures will be done at visit 3:

- ID is checked
- The participant returns any remaining doses. The number of remaining doses are noted and the participant's dosing document is collected.

- Any changes in medication are reported.
- Adverse events are noted, and appropriate follow-up is initiated.
- Any remaining capsules are returned for destruction.
- Participants leave their stool sample
- Length, weight, and waist-hip circumference are measured.
- Venous fasting blood samples are drawn for analysis of fasting blood glucose, HbA1c, insulin, c-peptide, Hb, GT, lipid levels (HDL, LDL, total cholesterol, triglycerides), TSH and free fatty acids. Total blood volume is estimated to 25 mL.
- Participants complete a questionnaire on dietary habits, physical activity, smoking and alcohol habits.
- A note is made in the medical record about the visit.
- Study is finished for the participant.

3.8 Questionnaire

The questionnaire at visit 2 and 3 will assess the following:

- Physical activity using the short IPAQ (international physical activity questionnaire), which is a commonly used scale to assess physical activity.
- Dietary habits using items that have been validated in Stockholm health questionnaire 2010 (Stockholms folkhälsoenkät 2010).
- Tobacco and alcohol, by validated items previously used by the national Health questionnaires in Sweden (Folkhälsoinstitutets nationella folkhälsoenkäter).

3.9 Number of participants

100 participants will participate in the study; 50 will receive BSE and 50 placebo.

3.10 Time plan

Aug 2018-Dec 2020

4. STUDY VARIABLES

4.1 Primary objective

The primary objective with the study is to test the hypothesis that BSE with standardized amounts (at least 150 µmole per daily intake) of sulforaphane, improves fasting blood glucose using intraindividual comparisons before and after treatment in the BSE relative to the placebo group.

4.2 Secondary objectives

The secondary objectives are to study the effect of BSE on the secondary effect variables, HbA1c, fatty liver index, fasting lipids, free fatty acids, insulin secretion, HOMA-IR and weight using intraindividual comparisons before and after treatment in the BSE relative to the placebo group.

4.3 Effect variables

The primary effect variable:

Venous fasting blood glucose. Venous fasting blood glucose will be analysed at screening visit (only participants having capillary glucose 6.0-6.9 mmol/l), visit 2 and 3 and used for effect analyses.

Secondary effect variables include:

- HbA1c
- a fatty liver index based on BMI, waist circumference, triglycerides and GT (Gastaldelli et al., 2009)
- fasting lipids, free fatty acids, insulin secretion
- HOMA-IR, based on fasting glucose and insulin
- Weight
- We will later analyse stool samples to investigate changes in abundance of bacterial strains between the BSE and placebo groups

5. STUDY COMPOUND

5.1 BSE

The study product is BSE with standardized amounts of sulforaphane. BSE contains a mixture of maltodextrin as a bulking agent and copper chlorophyllin (E 141) as a food additive. BSE is a dried powder of an aqueous extract of broccoli sprouts that provides a consistent and stable source of sulforaphane. A mixture of maltodextrin and copper chlorophyllin will be used as placebo. The active compound and the placebo are the same except BSE. The placebo will look similar to the BSE-containing mixture. Placebo or BSE powder will be provided as dry mixtures in sealed, non-transparent portion size bags. Each BSE bag contains 150 µmole, equal to 0.26 g, sulforaphane at a minimum. The mixtures are suspended with appr. 1 dl water and are ingested once daily in the morning. BSE should be stored at room temperature in dry conditions.

The doses of sulforaphane contained in the BSE is not possible to get by eating fresh broccoli, thus it has to be given as an extract.

5.2 Packing

Lantmännen will provide the dry mixtures with BSE or placebo as portion size bags. The bags are non-transparent and look similar between BSE and placebo. The mixtures will be ready to mix with liquid (e.g. water).

5.3 Randomization

Lantmännen will manufacture 60 kits with BSE and 60 with placebo and send to the assigned pharmacy in Göteborg. These kits will be divided into BSE or placebo so that it is obvious for the pharmacy what each kit contains. The randomization will be organized via Gothia Forum using a computer-based block randomization algorithm. Gothia Forum will send the randomization list in sealed envelopes to the local pharmacy, where each kit will then be marked with a number from 1 to 120 (corresponding to the study IDs). The local pharmacy will ensure that the kit marked 1 is BSE or placebo based on the randomization list.

Each participant receives the kit that corresponds to his/her study ID. The randomization is blind to both participants and the Investigator during the study. After the study, the participants have the right to know, upon asking, whether they received BSE or placebo.

The randomization list is kept with the local pharmacy during the entire study. Sealed envelopes with randomization information for each participant are send to investigator and may be opened in case of emergency.

The randomization list will be sent to A Rosengren after the study to enable data analysis.

5.4 Compliance

The participants should return any remaining capsules at visit 3. Only participants who have taken >75% of their capsules during the total study period and >80% during the last month will be included in the per protocol analyses. This is calculated by (the number of delivered bags – number of returned bags) / number of days in the study.

5.5 Reimbursement

Participants will receive travel reimbursement. This amount is subject to tax. Participants will receive the payment into their bank account at the end of the study.

6. STATISTICS AND DATA HANDLING

6.1 Statistics

The primary effect variable is venous fasting blood glucose. Participants will be analysed using intraindividual one-tailed comparisons before and after treatment and compared between the placebo and BSE arms using Student's t-test. The study hypothesis is that participants taking BSE for 12 weeks will have at least 0.3 mM lower fasting blood glucose on average than participants taking placebo. The intra-individual standard deviation of fasting blood glucose is 0.63 mM, based on analyses in our longitudinal cohorts of subjects with impaired fasting blood glucose. We therefore need 37 participants in each randomization group to detect a difference of 0.3 mM in fasting blood glucose between the groups at 80% power at $p<0.05$. We aim to recruit 50 participants in each group to cover for potential dropouts.

We will also make a subgroup analysis of participants with fasting triglycerides above median. This is based on our previous study on BSE (Axelsson et al.) showing that BSE is particularly effective in individuals with increased triglyceride levels. It is known that increased triglycerides are more common in subjects with elevated fatty liver content (Gastaldelli et al., 2009) and we therefore want to investigate whether this subgroup of the population would benefit more from BSE.

Both Intention-to treat (ITT) and per protocol (PP) analysis of the outcome variables will be performed and ITT will be used as a basis for the conclusions. Missing values will be replaced by the latest value available. Participants will poor compliance (see 5.4) will however not be included in any analyses.

The secondary study variables, HbA1c, fatty liver index, fasting lipids, free fatty acids, insulin, HOMA-IR and weight, will be analysed using intraindividual comparisons between visit 2 and 3.

We will later analyse stool samples to investigate changes in abundance of bacterial strains between the BSE and placebo groups.

6.2 Documentation / Case Report From

All data collected in the study will be registered in a case report form (CRF). The participant is identified through his/her study ID.

All AE are classified by the PI according to ICD10 and noted in the CRF.

The investigator, sponsor, monitor and persons assigned by the investigator have access to the CRF.

Data that are specific to the study and not relevant for the care of the participant do not have to be entered into the participant's medical record.

All data in the medical record must be in agreement with the CRF. The name of the study, the participant's study ID, date for informed consent and date for end of treatment must be noted in the medical record.

6.3 Data handling

Routine blood samples will be analysed at the central Clinical Chemistry laboratory, while some analyses (free fatty acids and stool samples) will be made using special kits at the University of Gothenburg. All data from CRF and blood analyses will be entered into a secure database. Only the sponsor or by him assigned persons will have access to the data. Only specific IT staff has physical access to the server facility. All stored data will be coded using study ID.

Study document and source data will be archived at least 10 years after study report.

Data will be published in peer-reviewed medical journals, at scientific conferences, for lay audience and in media. Only de-identified data based on averages will be used. Raw data on gene expression or microbiota have to be publically available at the Gene Expression Omnibus according to general rules in scientific journals. These data will be de-identified before publication.

The protocol may be published in part or full in accordance with scientific standard.

6.4 Monitoration

The study will be monitored by an independent monitor. It is the responsibility of the Investigator to ensure that the monitor has access to the CRF, the medical record and original laboratory data etc. to ensure that source data are relevant, without violating the integrity of the participants.

7. SAFETY

7.1 Safety of BSE

Cruciferous vegetables, including broccoli sprouts, are generally regarded as safe and are regular dietary components in many regions of the world.

Shapiro et al. 2006 conducted a double-blind, placebo controlled, randomized study of BSE in healthy individuals to determine the safety and tolerance of repeated oral administration. Twelve healthy human volunteers) received doses of BSE every 8 hours for 7 days (total 21 doses) while undergoing clinical evaluation and a battery of laboratory tests. Subjects received BSE corresponding to 75 – 300 μmol sulforaphane daily. No clinical adverse events were reported. Two of 12 individuals (both receiving BSE) showed an increase in plasma ALT exceeding the upper limit of normal. Notably, ALT levels rose for all subjects during the course of the study including placebo-treated subjects. Plasma AST levels rose above normal on day 19 for 2 of 12 subjects. Monitoring of TSH levels demonstrated that in 3 of 12 subjects (2 active treatments, 1 placebo), TSH levels exceeded the upper limit of normal during or after the dosing period. TSH increases were not associated with any clinical symptoms or abnormalities of T3 or T4. Evaluation by 2 independent endocrinologists determined that the changes in TSH were mild and reversible and did not pose an obstacle to further studies with BSE administration. No other significant laboratory abnormalities occurred. Thus, this safety study in healthy volunteers revealed no evidence of systematic, clinically significant adverse effects that could be attributed to the administration of BSE.

Kensler et al. 2005 conducted a human study enrolling 200 subjects. Healthy subjects were randomized to receive BSE containing 400 micromol sulforaphane (twice the dose we will use) or placebo nightly for 2 weeks. Subjects were followed clinically for compliance and adverse events. The BSE was well tolerated. 199 subjects completed all doses in the study without reporting any adverse events.

In a recent study of men with recurrent Prostate Cancer Alumkal et al. 2015 found that treatment with 200 micromol (the same dose that we will use) of sulforaphane per day for 20 weeks produced no major side effects.

The main potential AEs as reported previously might be expected to include gastrointestinal symptoms such as indigestion, belching, or loose stools.

Taken together, based on the above information we expect to see few if any adverse events associated with the administration of BSE. As with any food, an allergic reaction to broccoli sprouts is possible, though very unlikely.

7.2 Adverse events

Adverse event will be collected through spontaneously reporting and by telephone contact in between visits as well as by documentation at visit 3. Any unresolved AE at visit 3 will be followed up by referral or telephone contact. The telephone contact will be performed at latest within three weeks after Visit 3. All AE should be noted in the

CRF. If an AE requires hospital care the study code will be disclosed.

Any aberrant blood values discovered at the screening visit will not be considered as AEs and will be handled as appropriate, also for individuals who are not included.

Based on the widespread use of broccoli sprouts as a food and previous and ongoing human studies with BSE, no serious risks are anticipated.

All AE are documented in the CRF independent of possible relation to the treatment.

AEs are reported from visit 2 and serious adverse events (SAE) are reported from informed consent is obtained at visit 1.

8. ETHICAL ASPECTS

8.1 Risks - benefit

The study will be done in accordance with applicable laws and regulation and the principles of GCP and the Helsinki Declaration.

The benefits from participating exceed the risk in our opinion. Broccoli sprouts are used as a food supplement all over the world. BSE has been given in >30 clinical studies (www.clinicaltrials.gov). These studies have used BSE with up to 420 µmole sulforaphane (we will use 150 µmole) between 2 and 20 weeks (we will do 12 weeks treatment) and no serious adverse events or severe perturbations of routine blood analytes have been reported.

The only invasive procedures are the capillary and venous cannulations, which may give local pain.

There are several benefits for the participants, as they get a better characterization of their blood glucose levels and since we examine a number of blood analytes such as creatinine, liver status and lipid levels. We will follow up any abnormal values through referrals, including any suspected diabetes mellitus that is previously unknown.

If the treatment is effective it would provide a new means to lower blood glucose in the population that can serve as a "functional food".

8.2 Study information and ethical permits

The participants must give written informed consent before included in the study. The original of the consent form will be stored with the Investigator and the participants will receive a copy.

The participants will receive objective and neutral information about the study both in the invitation letter and at the study visits.

The study may start when there are approvals from the Ethical committee.

Substantial changes should be approved as Amendments by the Ethical committee.

All correspondence with the Ethical committee should be saved.

The study does not require permit from Läkemedelsverket, since it is testing a food supplement and does not aim to develop a drug. This issue has been discussed with Läkemedelsverket and they have stated that the study only needs approval by the local ethical committee since it is a food supplement study.

8.3 Insurance

The participants are insured through Patientskadelagen.

9. REFERENCES

Abdul-Ghani, M.A., Matsuda, M., Balas, B., and DeFronzo, R.A. (2007). Muscle and liver insulin resistance indexes derived from the oral glucose tolerance test. Diabetes Care 30, 89-94.

Alumkal et al. A phase II study of sulforaphane-rich broccoli sprout extracts in men with recurrent prostate cancer. Invest New Drugs. 2015 Apr;33(2):480-9.

AS Axelsson, E Tubbs, B Mecham, S Chacko, Y Tang, JW Fahey, JMJ Derry, CB Wollheim, N Wierup, MW Haymond, SH Friend, H Mulder & AH Rosengren. Sulforaphane reduces hepatic glucose production and improves glucose control in patients with type 2 diabetes Science Translational Medicine, 9, eaah4477. 2017.

Gastaldelli, A., Harrison, S.A., Belfort-Aguilar, R., Hardies, L.J., Balas, B., Schenker, S., and Cusi, K. (2009). Importance of changes in adipose tissue insulin resistance to histological response during thiazolidinedione treatment of patients with nonalcoholic steatohepatitis. Hepatology 50, 1087-1093.

Kensler, T. W., et al. (2012). "Modulation of the metabolism of airborne pollutants by glucoraphanin-rich and sulforaphane-rich broccoli sprout beverages in Qidong, China." Carcinogenesis **33**(1): 101-107.

Kensler TW, Chen J-G, Egner PA, Fahey JW, Jacobson LP, Stephenson KK, Ye L, Coady JL, Wang J-B, Wu Y, Sun Y, Zhang Q-N, Zhang B-C, Zhu Y-R, Qian G-S, Carmella SG, Hecht SS, Benning L, Ganage SJ, Groopman JD, Talalay, P (2005) *Effects of glucosinolate-rich broccoli sprouts on urinary levels of aflatoxin-DNA adducts and phenanthrene tetraols in a randomized clinical trial in He Zuo township, Qidong, People's Republic of China.* Cancer Epidemiol Biomarkers Prev 14: 2605-2613.

Lamb, J., et al. (2006). "The Connectivity Map: using gene-expression signatures to connect small molecules, genes, and disease." Science **313**(5795): 1929-1935.

Mari, A., Pacini, G., Murphy, E., Ludvik, B., and Nolan, J.J. (2001). A model-based method for assessing insulin sensitivity from the oral glucose tolerance test. Diabetes Care 24, 539-548.

Mari, A., Schmitz, O., Gastaldelli, A., Oestergaard, T., Nyholm, B., and Ferrannini, E. (2002). Meal and oral glucose tests for assessment of beta -cell function: modeling analysis in normal subjects. Am J Physiol Endocrinol Metab 283, E1159-1166.

Ravasz, E., et al. (2002). "Hierarchical organization of modularity in metabolic networks." Science **297**(5586): 1551-1555.

Shapiro TA, Fahey JW, Dinkova-Kostova AT, Holtzclaw WD, Stephenson KK, Wade KL, Ye L, Talalaly P (2006) *Safety, tolerance, and metabolism of broccoli sprout glucosinolates and isothiocyanates: A clinical Phase I study.* Nutr Cancer 55: 53-62.
